# Current treatment in macrophage activation syndrome worldwide: a systematic literature review to inform the METAPHOR project

**DOI:** 10.1093/rheumatology/keae391

**Published:** 2024-07-26

**Authors:** Francesco Baldo, Remco G A Erkens, Mao Mizuta, Greta Rogani, Federica Lucioni, Claudia Bracaglia, Dirk Foell, Marco Gattorno, Marija Jelusic, Jordi Anton, Paul Brogan, Scott Canna, Shanmuganathan Chandrakasan, Randy Q Cron, Fabrizio De Benedetti, Alexei Grom, Merav Heshin-Bekenstein, AnnaCarin Horne, Raju Khubchandani, Seza Ozen, Pierre Quartier, Angelo Ravelli, Masaki Shimizu, Grant Schulert, Christiaan Scott, Rashmi Sinha, Nicolino Ruperto, Joost F Swart, Sebastiaan Vastert, Francesca Minoia, PReS MAS/sJIA Working Party and Paediatric Rheumatology International Trial Organization, Kristiina Aalto, Carlos Abud Mendoza, Awatif Abushhaiwia, Constantin Ailioaie, Jonathan Akikusa, Guzide Aksu, Nuray Aktay Ayaz, Ruqaiya Nasser Al Jashmi, Safiya Al-Abrawi, Maria Alessio, Ekaterina Alexeeva, Sulaiman M Al-Mayouf, Abdulaziz AlMutairi, Muatasem Alsuweiti, Rizqi Amalia, Iman Amin, Jordi Anton, Wineke Armbrust, Itziar Astigarraga, Sevcan Bakkaloglu, Reima Bakry, Ozge Basaran, Floricely Basulto May, Jigna Bathia, Cristina Battagliotti, Alexandre Belot, Stefan Berg, Karin Beutel, Blanca Bica, Isabel Bolt, Martin Boyadzhiev, Oksana Boyarchuk, Yaryna Boyko, Claudia Bracaglia, Luciana Breda, Karine Brochard, Paul Brogan, Jurgen Brunner, Segundo Bujan Rivas, Aurelia Calin, Joan Calzada, Marisol Camacho Lovillo, Scott Canna, Elvira Cannizzaro, Roberta Caorsi, Raffaella Carlomagno, Marco Cattalini, Camilla Celani, Kwai Yu Winnie Chan, Sharat Chandra, Shanmuganathan Chandrakasan, Chong U Chang, Vyacheslav Chasnyk, Iryna Chyzheuskaya, Coziana Ciurtin, Daniel Clemente Garulo, Alexis-Virgil Cochino, Alessandro Consolaro, Rita Consolini, Randy Cron, Carlos Henrique M da Silva, Fabrizio De Benedetti, Carmen De Cunto, Arianna De Matteis, Lien De Somer, Fatma Dedeoglu, Chellapandian Deepakbabu, Emanuela Del Giudice, Adriana Soraya Diaz Maldonado, Pavla Dolezalova, Frank Dressler, Marta Dzhus, Yasser El Miedany, Dalia El-Ghoneimy, Wolfgang Emminger, Sandra Enciso, Anne Estmann, Hala Etayari, Danielle Fair, Maria Fasshauer, Daniel Fassi, Enrique Faugier, Silvia Federici, Brian Feldman, Giovanni Filocamo, Berit Flato, Mark Fluchel, Dirk Foell, Lampros Fotis, Marijan Frkovic, Robert Fuhlbrigge, Carla Gaggiano, Alenka Gagro, Romina Gallizzi, Ana Luiza Garcia Cunha, Fernando Garcia-Rodriguez, Fernando García-Rodríguez, Marco Gattorno, Hermann Girschick, Mia Glerup, Lyudmila Grebenkina, Suparna Guha, Raul Gutierrez Suarez, Jaime Guzman Ramirez, Djohra Hadef, Mohamad Hamad Saied, Soad Hashad, Philip (Pinchas) Hashkes, Henrik Hasle, Julia Allyson Hays, Martina Held, Jan-Inge Henter, Cristina N Herrera, Merav Heshin-Bekenstein, Assunta Chi Hang Ho, Anna Carin Horne, Gerd Horneff, Jing-Long Huang, Markus Hufnagel, Jaanika Ilisson, Mohammad Imnul Islam, Viktoriia Ivanova, Kazushi Izawa, Ales Janda, Marija Jelusic, Daechul Jeong, Rohith Jesudas, Ankur Jindal, Karla Vanessa Jiron Mendiola, Sheila K Oliveira, Robin Kahn, Rita Kaposzta, Ozgur Kasapcopur, Maria Martha Katsicas, Akhila Kavirayani, Camille Keenan, Parichat Khaosut, Khulood Khawaja, Waleed Ahmed Salaheldeen Hassan Khedr, Raju Khubchandani, Katarzyna Kobusinska, Oya Koker, Isabelle Koné-Paut, Mikhail Kostik, Jasmin Kuemmerle-Deschner, Ashish Kumar, Paul La Rosée, Mabel Aurora Ladino Ramirez, Calin Lazar, Chongwei Lee, Hartwig Lehmann, Kai Lehmberg, Caifeng Li, Xiaoqing Li, Francesco Licciardi, Joana Lima, Zoref Lorenz, Hala Lotfy, Daniel J Lovell, Meiping Lu, Kristīne Lukjanoviča, Maria Cristina Maggio, Silvia Magni-Manzoni, Sheren esam maher Maher, Mahmoud Majeed, Despoina Maritsi, Rebecca Marsh, Giorgia Martini, Tania Nicole Masmas, Maria Vincenza Mastrolia, Angela Mauro, Fatemeh Feresteh Mehregan, Manel Mejbri, Isabelle Melki, Paivi Miettunen, Angela Miniaci, Francesca Minoia, Mao Mizuta, Rakesh Mondal, Davide Montin, Zoilo Morel Ayala, Juan Manuel Mosquera Angarita, Zaure Mukusheva, Charlotte Myrup, Eka Nakhutsrishvili, Ahmed Naqvi, Hidehiko Narazaki, Joao Nascimento, Carmen Navarrete, Ellen Berit Nordal, Ekemini A Ogbu, Benson Ogunjimi, Lawrence Owino Okong'o, Filipa Oliveira-Ramos, Alessia Omenetti, Violetta Opoka-Winiarska, Francesca Orlando, Sumeyra Ozdemir Cicek, Seza Ozen, Clare Pain, Priyankar Pal, Natalia Palmou Fontana, Charalampia Papadopoulou, Manuela Pardeo, Gordana Petrovic, Mercedes Picarelli, Rakesh Kumar Pilania, Clarissa Pilkington, Maria del Carmen Pinedo, Polyxeni Pratsidou-Gertsi, Chris Pruunsild, Maa-Ohui Quarmyne, Pierre Quartier, Seyed Reza Raees Karami, Kim Ramme, Angelo Ravelli, Jerome Razanamahery, Katariina Rebane, Agustin Remesal, Karine Retornaz, Donato Rigante, Joseph Rocco, Adriana Rodrigues Fonseca, Ana Luisa Rodriguez Lozano, Sandra Rodriguez-Aguayo, Jorge Rojas, Martina Rossano, Samppa Ryhanen, Claudia Saad Magalhaes, Payman Sadeghi, Erdal Sag, Blachy Javier Saldana Davila, Farhad Salehzadeh, Judith Sánchez-Manubens, Sujata Sawhney, Grant Schulert, Adrien Schvartz, Yuksel Selcuk, Velma Selmanovic Mulaosmanovic, Ethan Sen, Seher Sener, Rachna Shanbhag Mohite, Avinash Sharma, Rawia Salama Shehata, Masaki Shimizu, Gabriele Simonini, Surjit Singh, Roubini Smerla, Aušra Šnipaitienė, Ali Sobh, Betul Sozeri, Mihaela Sparchez, Saša Sršen, Valda Stanevicha, Joost Swart, Flavio Sztajnbok, Sirikarn Tangcheewinsirikul, Katya Temelkova, Klaus Tenbrock, Natasa Toplak, Lilibeth Torno, Matteo Trevisan, Maria Tsinti, Elena Tsitsami, Marinka Twilt, J Merlijn Van den Berg, Jan A M van Laar, Camilo Andres Vargas Rincon, Giulia Camilla Varnier, Sebastiaan Vastert, Gabriel Vega Cornejo, Lucio Verdoni, Diego Oscar Viola, Jelena Vojinovic, Yulia Vyzhga, Bjorn Wahlin, Hiroyuki Wakiguchi, Peter Weiser, Ewa Wiesik-Szewczyk, Kazuko Yamazaki, Junko Yasumura, Wei Yin, Huasong Zeng, Wei Zhang, Vahid Ziaee, Amit Ziv, Zbigniew Zuber

**Affiliations:** Pediatric Immuno-Rheumatology Unit, Fondazione IRCCS Ca’ Granda Ospedale Maggiore Policlinico, Milan, Italy; ASST Gaetano Pini, Milan, Italy; Department of Pediatric Rheumatology and Immunology, University Medical Center Utrecht, Utrecht, the Netherlands; Department of Pediatric Rheumatology, Hyogo Prefectural Kobe Children's Hospital, Kobe, Japan; Department of Pediatric Rheumatology and Immunology, University Medical Center Utrecht, Utrecht, the Netherlands; Pediatric Immuno-Rheumatology Unit, Fondazione IRCCS Ca’ Granda Ospedale Maggiore Policlinico, Milan, Italy; Division of Rheumatology, IRCCS Ospedale Pediatrico Bambino Gesù, Rome, Italy; University Hospital Muenster, Muenster, Germany; Reumatologia e Malattie Autoinfiammatorie, IRCCS Istituto Giannina Gaslini, Genoa, Italy; University Hospital Centre Zagreb, University School of Medicine, Zagreb, Croatia; Hospital Sant Joan de Déu, Universitat de Barcelona, Barcelona, Spain; Great Ormond Street Hospital for Children, London, UK; University College London Institute of Child Health, London, UK; Children’s Hospital of Philadelphia, Philadelphia, PA, USA; Aflac Cancer and Blood Disorders Center Children’s Healthcare of Atlanta, Emory University School of Medicine, Atlanta, GA, USA; University of Alabama at Birmingham, Birmingham, AL, USA; Division of Rheumatology, IRCCS Ospedale Pediatrico Bambino Gesù, Rome, Italy; Cincinnati Children’s Hospital, Cincinnati, OH, USA; Dana Dwek Children's Hospital, Tel Aviv Medical Center, Tel Aviv University, Tel Aviv, Israel; Department of Pediatrics, Karolinska University Hospital, Stockholm, Sweden; Department of Women’s and Children’s Health, Karolinska Institute, Stockholm, Sweden; SRCC Childrens Hospital, Mumbai, India; Department of Pediatrics, Hacettepe University, Ankara, Turkey; Université Paris-Cité, Paris, France; RAISE Reference Centre, Pediatric Immunology-Hematology and Rheumatology Unit, Necker-Enfants Malades Hospital, Paris, France; Direzione Scientifica, IRCCS Istituto Giannina Gaslini, Genoa, Italy; Department of Pediatrics and Developmental Biology, Graduate School of Medical and Dental Sciences, Tokyo Medical and Dental University, Tokyo, Japan; Cincinnati Children’s Hospital, Cincinnati, OH, USA; University of Ottawa, Ottawa, Canada; Systemic JIA Foundation, Cincinnati, OH, USA; Gaslini Trial Centre/Servizio Sperimentazioni Cliniche Pediatriche, PRINTO, IRCCS Istituto Giannina Gaslini, Genoa, Italy; Department of Pediatric Rheumatology and Immunology, University Medical Center Utrecht, Utrecht, the Netherlands; Department of Pediatric Rheumatology and Immunology, University Medical Center Utrecht, Utrecht, the Netherlands; Pediatric Immuno-Rheumatology Unit, Fondazione IRCCS Ca’ Granda Ospedale Maggiore Policlinico, Milan, Italy

**Keywords:** macrophage activation syndrome, haemophagocytic syndromes, haemophagocytic lymphohistiocytosis, treatment

## Abstract

**Objective:**

To assess current treatment in macrophage activation syndrome (MAS) worldwide and to highlight any areas of major heterogeneity of practice.

**Methods:**

A systematic literature search was performed in both EMBASE and PubMed databases. Paper screening was done by two independent teams based on agreed criteria. Data extraction was standardized following the PICO framework. A panel of experts assessed paper validity, using the Joanna Briggs Institute appraisal tools and category of evidence (CoE) according to EULAR procedure.

**Results:**

Fifty-seven papers were finally included (80% retrospective case-series), describing 1148 patients with MAS: 889 systemic juvenile idiopathic arthritis (sJIA), 137 systemic lupus erythematosus (SLE), 69 Kawasaki disease (KD) and 53 other rheumatological conditions. Fourteen and 11 studies specified data on MAS associated to SLE and KD, respectively. All papers mentioned glucocorticoids (GCs), mostly methylprednisolone and prednisolone (90%); dexamethasone was used in 7% of patients. Ciclosporin was reported in a wide range of patients according to different cohorts. Anakinra was used in 179 MAS patients, with a favourable outcome in 83% of sJIA-MAS. Etoposide was described by 11 studies, mainly as part of HLH-94/04 protocol. Emapalumab was the only medication tested in a clinical trial in 14 sJIA-MAS, with 93% of MAS remission. Ruxolitinib was the most reported Janus kinase inhibitor in MAS.

**Conclusion:**

High-dose GCs together with IL-1 and IFNγ inhibitors have shown efficacy in MAS, especially in sJIA-associated MAS. However, the global level of evidence on MAS treatment, especially in other conditions, is still poor and requires standardized studies to be confirmed.

Rheumatology key messages

- High-dose GCs together with IL-1 and IFNγ inhibitors have shown efficacy in sJIA-associated MAS.
- Current level of evidence on MAS treatment, especially in condition other than sJIA, is still poor.
- MAS treatment is still extremely variable, with potential significant discrepancies across different centres and countries.

## Introduction

Macrophage activation syndrome (MAS) is an hyperinflammatory life-threatening condition, part of the wide spectrum of haemophagocytic lymphohistiocytosis (HLH). The term MAS refers to a secondary form of HLH that complicates the course of rheumatological conditions. MAS is characterized by a marked hyperferritinaemia, cytopenia, liver insufficiency with coagulopathy, neurological manifestations and a high risk of rapid progression to multiorgan failure. Despite great improvement in diagnosis and management [1–9], MAS still represents a major challenge in clinical practice.

MAS treatment remains largely empirical and based on expert consensus. Although promising data are emerging, results from large cohorts and standardized trials are still required for most medications used to treat MAS. Multinational data on systemic juvenile idiopathic arthritis (sJIA)-associated MAS highlighted several disparities in its management in relation to geographic location of the treating centre and subspecialty of the caring physicians [10]. Recently, the first international recommendations for the early-stage management of HLH/MAS have been published [11]. Despite their milestone relevance, these guidelines focus on the initial management of the spectrum of haemophagocytic syndromes and do not specifically address the treatment of MAS. Furthermore, there is a particular lack of evidence on the therapeutic approach to MAS associated with rheumatological conditions other than sJIA. It is thus conceivable that a wide heterogeneity in the management of MAS exists, due to differences in treatment strategies, access to medications and involvement of different specialists.

The METAPHOR project was conceived to provide an overview of current real-life therapeutic approaches to MAS in different clinical settings worldwide by means of a web-survey involving the paediatric rheumatology community part of the Pediatric Rheumatology European Society (PReS) and the Pediatric Rheumatology International Trial Organization (PRINTO) and the paediatric haematologists from the Histiocyte Society. In this context, a systematic literature review (SLR) to explore available data on MAS treatment was performed.

## Methods

**Figure 1.**
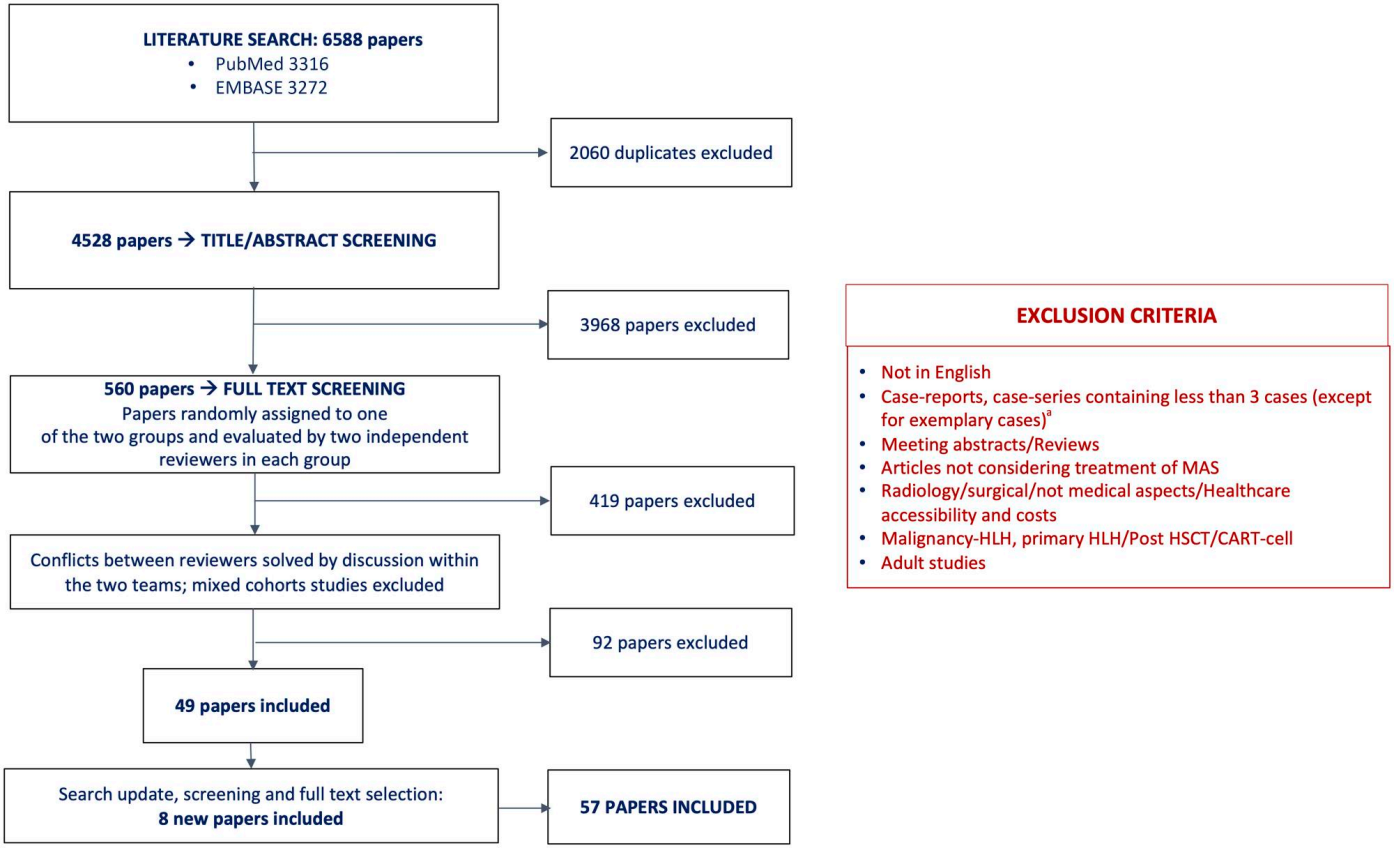
Flowchart for the systematic literature review, including detailed exclusion criteria, and results of the selection process. ^a^Seven case reports were exceptionally included after a discussion within the core team for the relevancy of the medication or the condition reported. CART-cell: chimeric antigen receptor T cell; HLH: haemophagocytic lymphohistiocytosis; HSCT: haematopoietic stem cell transplantation; MAS: macrophage activation syndrome

The SLR was conducted following the EULAR standardized operating procedures [12]. A multinational panel of experts in the field of MAS was involved. The PICO (Patient–Intervention–Comparison–Outcome) framework was adopted to structure the research (see Supplementary Data S1 and Supplementary Table S1, available at *Rheumatology* online). Acknowledging the concomitant international effort of the EULAR/PRES task force for sJIA and adult-onset Still’s disease, which includes a SLR on the treatment of sJIA-associated MAS (De Matteis *et al.* [13]), we decided to particularly address MAS in conditions other than sJIA. On 30 June 2022 the literature search was performed in both PubMed and EMBASE databases, and then updated on 30 June 2023. Search strings were designed under the supervision of an expert librarian (see Supplementary Text, available at *Rheumatology* online). Main inclusion criteria were: original articles, English language, studies reporting data regarding treatment of patients with MAS, population’s age <18 years old and papers with >3 cases reported. Exclusion criteria are detailed in Fig. 1. In light of the scarcity of available data on specific conditions or medication, and only after discussion in our core team, we did exceptionally include a case-report, if this was deemed relevant for the analysis. Papers were checked for duplicates and then screened, using Rayyan software (Cambridge, MA, USA). A first title and abstract screening was performed, and then selected papers were evaluated through a full-text read.

To establish the quality and the category of evidence of included papers, two members of the Expert Panel evaluated each manuscript independently. The Joanna Briggs Institute critical appraisal tools were used to assess the validity score [14], identifying three validity levels (low, moderate, high), and the category of evidence (CoE) was attributed as per EULAR standardized operating procedures [12].

## Results

A total of 6588 papers were identified through the first search. After the deletion of duplicates and the title/abstract selection, 560 articles underwent full text screening and finally 57 studies fulfilled the eligibility criteria (Fig. 1). Twenty-three papers reported sJIA cohorts, four systemic lupus erythematosus (SLE) cohorts, eight Kawasaki disease (KD) cohorts, while in 22 studies the described population was mixed. Thirty-six were single-centre retrospective case series, 10 multicentre retrospective case series, two single-centre retrospective cohorts, one multicentre prospective cohort, only one was a standardized single arm open label clinical trial; seven case reports were included for the relevancy of the medication or the condition reported. Three additional studies about Janus kinase (JAK) inhibitors (JAK-i) [15–17] were considered, despite reporting data about mixed HLH cohorts; data from those studies only contributed to the JAK-i evidence review. Most papers (84%) were found to have low or moderate validity, and almost all (96%) were classified with a CoE of 3 or 4. Supplementary Table S2, available at *Rheumatology* online, reports all the information available on papers included in the SLR.

Data from a total of 1148 patients with MAS were finally evaluated: 889 sJIA, 137 SLE, 69 KD and 53 other rheumatological conditions, including eight juvenile dermatomyositis, seven mixed connective tissue disease, six vasculitis, two antiphospholipid syndrome, two spondyloarthritis, two undefined connective tissue disease, two polyarticular JIA, one undefined arthritis, one rheumatic fever, one enthesitis-arthritis (ERA), one Kikuchi disease, one Sjögren disease, one sarcoidosis, one cryopyrin associated periodic syndrome, one mevalonate-kinase deficiency (MKD), one Crohn’s disease and 15 unspecified rheumatic disorders.

### Glucocorticoids

All studies mentioned the use of GCs and information was available for 1054 MAS patients (829 sJIA, 91 SLE, 66 KD, 68 other rheumatological conditions). Among the 300 patients in which this information was assessable, most patients (86%, 258/300) received GCs as a co-medication, while 42/300 (14%) were successfully treated with GCs as monotherapy. Methylprednisolone (MPN) or prednisolone were the mostly used GCs (90%), followed by dexamethasone (DEX, 7%). DEX was used in 15%, 10% and 6% of patients with MAS in the context of KD, SLE and sJIA, respectively.

MPN dose ranged from 2 to 30 mg/kg/day, with high-dose MPN pulses (10–30 mg/kg/day) reported in almost 60% of studies. Interestingly, a tapering regimen of MPN pulses was suggested by Loganathan *et al.* for severe MAS complicating sJIA in a resource limited setting [18]. DEX dose ranged from 4 to 10–15 mg/m^2^/day. Two Japanese studies [19, 20], reported the successful use of dexamethasone palmitate (DEX-P), a liposomal incorporated formulation, in 24 sJIA-MAS patients (17 naïve and seven refractory to MPN/prednisolone ± ciclosporin [CsA]).

### Ciclosporin

Fifty studies mentioned the use of CsA in 611 MAS patients (483 sJIA, 34 SLE, 10 KD, 84 other rheumatological diseases). In the largest multinational cohort of sJIA-MAS [21], CsA was the medication most frequently prescribed besides GCs (61% of patients). Only 10 studies reported details about the route and the dose of administration: CsA was given intravenously (i.v.) in 29 patients and orally in 12, with dose ranging from 0.8 to 8 mg/kg/day. Trough levels were mentioned only in three studies [22–24] and ranged between 78 and 480 ng/ml.

Globally, outcome in patients treated with CsA was assessable for 186 patients (138 sJIA, 9 SLE, 8 KD, 31 other rheumatic diseases): in six patients (3%) a poor outcome (four deaths, two severe neurological adverse events) was reported. Posterior reversible encephalopathy syndrome (PRES) was mentioned in one sJIA-MAS patient, who was receiving co-treatment with GCs, IVIG and etoposide [25]. Five sJIA-MAS patients were successfully treated with CsA without modification of the background GC therapy [23, 26].

### Etoposide

Details on etoposide were available from 11 studies, for a total of 120 patients (78 SJIA, 14 SLE, 14 KD, 14 other rheumatic diseases); outcome data were available for 17 sJIA, 7 SLE, 14 KD and 4 other rheumatic diseases. Seven patients (17%) died. Neutropenia was the main adverse event reported; in three patients, severe bone marrow suppression with sepsis was reported.

Dose of etoposide ranged from 50 to 150 mg/m^2^ weekly–biweekly. Of note, two studies reported the use of low dose etoposide (50–100 mg/m^2^/week for 4–11 weeks) [27, 28], in seven patients with MAS (five sJIA and two SLE). All sJIA patients were refractory to high-dose GCs and CsA, 3/5 also to anakinra (2.7–15 mg/kg/day), and all achieved MAS remission after etoposide. The two patients with SLE had failed oral prednisone: both survived with MAS remission, but one developed long-term CNS sequela.

### Anakinra

A total of 179 patients received anakinra for MAS (147 sJIA, 12 SLE, 1 KD, 19 other rheumatological disorders), reported in 19 studies all published after 2011. Outcome data were available for 82 sJIA, 10 SLE, 1 KD, 12 other rheumatological conditions and for three secondary HLH (sHLH) treated with i.v. anakinra continuous infusion (Table 1). A complete response was reported in 68 patients with sJIA-MAS (83%); eight patients presented an incomplete (10%) and three (4%) a lack of response to anakinra, two had a recurrency of MAS and two (2%) died. Patients with SLE-MAS treated with anakinra had a favourable outcome in 6/10 cases (60%), with four reported deaths (40%).

**Table 1.** Data available on patients with MAS treated with anakinra

First author, year [ref]	Type of publication	Population	Pts treated with ANK	**ANK dose/route of administration**	Previous treatments for MAS	Other treatments	Outcome	Validity score, EULAR CoE
Miettunen PM, 2011 [29]	Retrospective case series	12 MAS (8 sJIA, 2 AAV, 1 KD, 1 ARF)	12/12	2 mg/kg/day s.c. (max 100 mg/day) once daily	MPN (100%), IVIG (75%), CsA (83%), etoposide (16%), antiTNF (8%)	Etoposide, anti TNF stopped; all other treatments continued	12/12 CR (median time to remission: 13 days)	Moderate, 3
Bennett TD, 2012 [30]	Retrospective case series	102 JIA (90 sJIA) 19 SLE	15 JIA-MAS	NA	NA	GCs (93%), CsA (33%), etoposide (7%)	NA	Moderate, 3
Minoia F, 2014 [21]	Retrospective case series	362 sJIA-MAS	33 sJIA-MAS	NA	NA	GCs (98%), CsA (61%), IVIG (36%), etoposide (12%)^a^	NA	High, 3
Ozturk K, 2015 [31]	Case report	1 sJIA-MAS	1 sJIA-MAS	2 mg/kg/day	MPN, DEX, etoposide, CsA, tacrolimus	ATG	1/1 CR	Low, 4
Barut K, 2015 [32]	Retrospective case series	10 sJIA-MAS	5 sJIA-MAS	NA	NA	GCs (100%), CsA (80%), CNK (40%)^a^	NA	Low, 3
Aytaç S, 2016 [33]	Retrospective case series	31 sJIA-MAS 6 SLE-MAS	13 sJIA-MAS 2 SLE-MAS	NA	NA	GCs (100%), IVIG (68% sJIA, 33% SLE), CsA (74% sJIA 68% SLE), etoposide (32% sJIA, 50% SLE)	11/13 sJIA-MAS CR	Moderate, 3
Silva JMF, 2018 [34]	Retrospective case series	16 refractory JIA (4 sJIA-MAS)	4 sJIA-MAS	NA	NA	3 pts HSCT for refractory MAS, 1 pt developed MAS after HSCT GCs (100%), CsA (100%), etoposide (25%), ATG (25%)	3/4 CR 1/4 died	Moderate, 3
Borgia RE, 2018 [35]	Retrospective cohort	38 SLE-MAS	2 SLE-MAS	NA	NA	GCs (100%), IVIG (58%), CsA (29%), etoposide (13%)^a^ 2/2 pts treated with ANK received PE, 1/2 intrathecal MTX, 1 alemtuzumab	2/2 death	High, 3
Sönmez HE, 2018 [36]	Retrospective case series	15 sJIA, 2 AID (19 MAS episodes)	19/19	2–6 mg/kg/day	All pts received ANK as first line	GCs (100%), CsA (63%), etoposide (16%), IVIG (% not reported)	13/15 sJIA CR 2/15 sJIA recurrent MAS	Moderate, 3
Eloseily EM, 2020 [37]	Retrospective case series	28 MAS (13 sJIA, 5 SLE, 3 MCTD, 7 others) 16 sHLH (3 malignancies)	44/44	sJIA: 2.9–11.9 mg/kg/day SLE/MCTD: 2–48 mg/kg/day (latter as continuous i.v. infusion).	NA	sJIA: GCs (54%), CsA (23%) SLE/MTCD: GCs (87%), CYC (13%)	13/13 sJIA-MAS CR 2/5 SLE death	Moderate, 3
Charlesworth JEG, 2021 [38]	Case report	2 sHLH	2/2	Pt1: 12 mg/kg/day → 48 mg/kg/day Pt2: 11 mg/kg/day 2/2 received continuous i.v. infusion	2/2: MPN, IVIG	Pt1: etoposide (1 dose), CsA	2/2 CR	High, 4
Phadke O, 2021 [39]	Retrospective case series	14 MAS (10 sJIA, 3 SLE, 1 vasculitis) 5 sHLH	19/19	Initial dose: 1.7–10 mg/kg/day i.v. Max. dose: 4.2–15.4 mg/kg/day i.v. (max 400 mg/day)	NA	NA	No SAE reported 1/10 sJIA-MAS died (MPN, DXA, VP16, JAK-i) for sepsis 1/1 vasculitis-MAS died (CYC, RTX, ECZ) with stroke and MOF	Moderate, 3
Horne AC, 2021 [27]	Retrospective case series	7 MAS (5 sJIA, 2 SLE)	3 sJIA-MAS	2.7–15 mg/kg/day	NA	3/3: GCs, CsA, low-dose etoposide 1/3: IVIG	3/3 no response, requiring low dose etoposide (2/3 discontinued ANK)	Moderate, 3
Minoia F, 2021 [40]	Retrospective case series	23 MAS-TMA (17 sJIA, 2 SLE, 1 JDM, 1 MCTD, 2 UCTD)	10 MAS (7 sJIA)	NA	NA	GCs (100%), CsA (61%, 12 sJIA), IVIG (74%, 13 sJIA), etoposide (17%, 4/4 sJIA) PE (74%, 11 sJIA), ECZ (39%, 4 sJIA), RTX (26%, 3 sJIA)^a^	NA	High, 3
Aydin F, 2021 [41]	Retrospective case series	7 sJIA-MAS	4 sJIA-MAS	NA	NA	GCs (100%), CNK (75%), CsA (50%), IVIG (25%)	3/4 CR 1/4 death (GCs, CNK)	Low, 3
Bağlan E, 2022 [42]	Retrospective cohort	10 sJIA-MAS	5 sJIA-MAS	NA	NA	GCs (100%), IVIG + PE (80%), CsA (10%), TCZ (10%)^a^	NA	Moderate, 3
De Benedetti F, 2023 [43]	Controlled clinical trial	14 sJIA-MAS	7 sJIA-MAS	1.6–15 mg/kg/day	NA	GCs (100%), CsA (57%), IVIG (21%)^a^ All patients treated with emapalumab	Incomplete response, requiring emapalumab (2/7 discontinued ANK)	High, 2A
Chellapandian N, 2023 [44]	Case report	1 refractory sJIA-LD, recurrent MAS	1/1	2–4 mg/kg/day	NA	MPN, CsA, CNK, TCZ Emapalumab added on top of ANK, HSCT	Incomplete response, requiring emapalumab and HSCT	High, 4
Rossano M, 2023 [45]	Retrospective case series	14 MAS (6 sJIA, 3 SLE, 2 JDM, 3 unknown)	3 sJIA-MAS	5 mg/kg/day	NA	3/3: MPN, CsA	3/3 CR	Moderate, 3

a Data refer to the overall population included in the study and not specific for patient treated with anakinra. AAV: ANCA-associated vasculitis; AID: autoinflammatory disease; ANK: anakinra; ARF: acute rheumatic fever; ATG: anti-thymocyte globulin; CNK: canakinumab; CoE: category of evidence; CsA: ciclosporin A; CR: complete remission; CYC: cyclophosphamide; DEX: dexamethasone; ECZ: eculizumab; GCs: glucocorticoids; i.v.: intravenous; HSCT: hematopoietic stem cell transplant; IVIG: intravenous immunoglobulin; JAK-i: Janus kinase inhibitor; JDM: juvenile dermatomyositis; KD: Kawasaki disease; LD: lung disease; MAS: macrophage activation syndrome; MPN: methylprednisolone; MCTD: mixed connective tissue disease; MOF: multiorgan failure; MTX: methotrexate; NA: not available; PDN: prednisone; PE: plasma exchange; pt: patient; RTX: rituximab; SAE: severe adverse event; s.c.: subcutaneous; sHLH: secondary haemophagocytic lymphohistiocytosis; sJIA: systemic juvenile idiopathic arthritis; SLE: systemic lupus erythematosus; TCZ: tocilizumab; TMA: thrombotic microangiopathy; UCTD: undifferentiated connective tissue disease.

In the included studies, anakinra was used with a wide dosing range (2–48 mg/kg/day). The highest dose was used as continuous i.v. infusion in two patients: one patient with MAS secondary to SLE/MCTD was treated for 72 h without any other medication, but eventually died from multiorgan failure [37]. The second patient was a 9-year-old girl with severe sHLH and neurological involvement without a known trigger, refractory to MPN pulses and IVIG and anakinra (12 mg/kg/day); given her worsening conditions, anakinra was steeply increased to 2 mg/kg/h (48 mg/kg/day) with a positive outcome [38]. The use of high-dose anakinra (at least 5 mg/kg/day) was specified in six studies [27, 36–38, 43, 45] for 27 patients, and 93% of them were reported after 2020.

Concomitant medications in patients treated with anakinra were assessable only for 67 episodes of MAS. High-dose anakinra was reported mainly together with GCs and CsA (85% and 37%, respectively), followed by etoposide (15%). Anakinra was used as monotherapy in six patients (five sJIA and one SLE/MTCD) [37]: all patients with sJIA achieved MAS remission (dosing range of 2.9–6.2 mg/kg/day), while the patient with SLE/MTCD died despite being treated with high doses (48 mg/kg/day i.v.). Data on MAS patients treated with anakinra as single medication on the background of GCs were available from two studies [36, 37] reporting 15 episodes of MAS: all the 10 episodes with assessable outcome data achieved MAS remission.

### Emapalumab

The first and only clinical trial in MAS assessed the role of emapalumab (anti-IFNγ monoclonal antibody) on sJIA-associated MAS refractory to high-dose GCs [43]. In this single-arm, open label trial, 14 sJIA-MAS were included: eight were refractory also to CsA and seven to anakinra. By week 8, MAS remission was achieved in 13/14 patients (93%), with a median time to remission of 25 days. In all patients, emapalumab led to a rapid regression of all MAS parameters and to a significant steroid-sparing effect. No deaths or serious adverse events related to emapalumab were reported. Viral infection/seropositivity was the most frequent side effect (mainly CMV; of note, all patients received acyclovir prophylaxis). Interestingly, the combination of emapalumab with anakinra (up to 4 mg/kg/day) seemed to reduce the occurrence of sJIA flare without increasing serious events and infection rate. In the trial one patient received emapalumab together with high-dose anakinra (7.5 mg/kg/day), with good tolerability and without the mention of specific adverse events.

### Other biologics

The use of other biologics in the treatment of MAS was reported in 22 studies: canakinumab and tocilizumab were the most commonly reported biologic agents for sJIA-MAS, while infliximab was mainly used in patients with KD-MAS (seven patients treated with a dose range 3–10 mg/kg/day and a positive outcome).

Thirty-five patients [34, 46–49] received tocilizumab, and in 26 of them outcome data were available: 22 patients (85%) had MAS remission; in one tocilizumab was discontinued for lack of response (4%) and in three (12%) for an allergic reaction. Of note, in the two main cohorts of sJIA-MAS patients successfully treated with TCZ [46, 48], none of them previously received an IL-1 inhibitor.

Canakinumab was used in 16 patients [32, 41, 49, 50], with a positive response in 14 of them (88%). In particular, Kostik *et al.* [49] described eight sJIA-MAS patients all treated with canakinumab: seven achieved MAS remission and one required the addition of tofacitinib to control MAS recurrency. In three patients, canakinumab was successfully used as first line biologic treatment. Interestingly, three patients developed severe MAS despite canakinumab standard treatment, and responded to an increase of canakinumab dose, up to 12 mg/kg.

In a cohort of MAS associated to thrombotic microangiopathy (TMA) [40], nine patients received complement inhibition (eculizumab) in addition to MAS-target treatment: seven patients achieved regression of both MAS and TMA and two died.

### JAK inhibitors

In our SLR only one study reporting JAK-i was specifically focused on MAS [50]. In this paper, authors described 10 refractory sJIA, three of them with severe MAS resistant to high-dose GCs and tocilizumab (one also to etoposide). All of them were treated with ruxolitinib (2.5–5 mg × 2/day) with a rapid regression of MAS without adverse events. Notably, none received IL-1 inhibitors or CsA before JAK-i introduction, and all required the further addition of canakinumab to control underlying sJIA.

Three other studies [15–17] reported the use of ruxolitinib in mixed cohorts of sHLH patients. In a retrospective case series of nine patients (five EBV-HLH, two fHLH, one MAS, one unspecified) refractory to the HLH94 protocol, three patients (1 MAS) achieved MAS remission, while others required the association with DEX-P [15]. In a case–control study [16], 11 patients (including two sJIA-MAS and one KD-MAS) were successfully treated with ruxolitinib (seven refractory to HLH04 protocol, four naïve). In a pilot, open-label, single arm trial [17] 12 sHLH patients (eight EBV-HLH, two MAS, two unspecified) received ruxolitinib as first line treatment with a positive response in 10 of them.

The only other JAK-i mentioned as a treatment for sJIA-MAS was tofacitinib in two patients: in one case tofacitinib was ineffective and was switched to ruxolitinib [50], while in the other it contributed to control MAS recurrency together with canakinumab [49].

### Haematopoietic stem cell transplantation

Six studies reported data about haematopoietic stem cell transplantation (HSCT) in patients with refractory MAS [24, 30, 34, 44, 47, 51]. In a case series Silva *et al.* [34] described five patients with refractory sJIA-MAS treated with allogeneic HSCT: one patient died from pulmonary haemorrhage 85 days after HSCT, three developed graft *vs* host disease and 5/5 had severe infections following HSCT. All but one patient developed 100% chimerism, and all patients who survived achieved disease remission after HSCT. Chellapandian *et al.* [44] described a 4-year-old child with sJIA, recurrent MAS and lung disease, refractory to GCs, anakinra, methotrexate, tocilizumab and canakinumab, who was successfully treated with emapalumab as bridge therapy to a matched sibling donor allogenic HSCT. HSCT was further mentioned in four MAS and four sHLH [24, 30, 47, 51]: outcome data were available for two MAS, who survived without disease reactivation, and for sHLH patients, of whom one died.

### Other treatments

Use of IVIG was reported in 280 sJIA, 46 SLE, 37 KD and 48 other rheumatic diseases, from 41 studies. However, specific data on IVIG efficacy are extremely hard to extract, as IVIG was almost always used as part of a combined regimen and no studies focused on IVIG efficacy were found. In 15 studies, plasma-exchange (PE) was mentioned as additional treatment for MAS. Overall, 48 patients with sJIA, nine with SLE and six with other rheumatic diseases received PE for MAS. In particular, PE was used as part of a combination therapy in 17 patients to control MAS-associated TMA [40].

### Treatment of MAS in rheumatological diseases other than sJIA

Fourteen papers presented detailed data about SLE-MAS, for a total of 105 patients, with an overall mortality of 7% (Table 2). Bennett *et al.* [30] compared the differences in MAS treatment between SLE and sJIA in a cohort of 102 sJIA and 19 SLE. SLE patients were more frequently given DEX (32% *vs* 14%, *P* = 0.05), cyclophosphamide (21% *vs* 3%, *P* = 0.01) and MMF (32% *vs* 2%, *P* < 0.001); only children with underlying sJIA received IL-1 antagonists. Similarly, in the cohort by Aytaç *et al.* [33], all patients with sJIA seen after 2011 received anakinra, while patients with SLE were treated more frequently with IVIG (68% *vs* 33%) and etoposide (50% *vs* 32%), and received IL-1 blockade in 30% of cases. In the large cohort of SLE-MAS described by Borgia *et al.* [35], only two patients were treated with anakinra: both patients were refractory to several treatments, including PE and in one case alemtuzumab and intrathecal methotrexate, and eventually died.

**Table 2.** Treatment data available on patients with SLE-associated MAS

First author, year (ref)	Type of publication	Country	Pts with SLE-MAS	MAS prevalence	Treatment	Outcome	Validity score, EULAR CoE
Cortis E, 2006 [52]	Retrospective case series	Italy	1	NA	MPN pulses + CsA	Remission	Low, 3
Lambotte O, 2006 [53]	Retrospective case series	France	12 (15 episodes)	1.0%	14/15 GCs (9 MPN + PDN, 3 PDN); 2/15 oral PDN in monotherapy; 6/15 IVIG (5/6 as first line, 3/6 first line monotherapy); 2/15 CYC (1 after failure of etoposide + CsA and RTX) 1 pt without specific treatment	Patient without specific treatment relapsed → MPN; 3/3 IVIG monotherapy did not respond → GCs; 5/15 ICU	Moderate, 3
Islam MI, 2017 [54]	Retrospective case series	Bangladesh	2	NA	MPN, followed by oral PDN	NA	Low, 3
Bennett TD, 2012 [30]	Retrospective case series	US	19	NA	19/19 GCs (6/19 DEX); 8/19 CsA alone, 1/19 etoposide + 1 VP16 and CsA; 7/19 IVIG; 2/19 PE; 6/19 MMF; 2/19 RTX	12/19 (63%) ICU; 2/19 (11%) mortality	Moderate, 3
Gokce M, 2012 [55]	Retrospective case series	Turkey	6	NA	6/6 CS (3 MPN, 3 DEX); 3/6 HLH-2004 protocol; 3/6 CsA + IVIG; 2/6 PE (TMA)	1/6 (16% mortality) treated with HLH-2004 protocol	Low, 3
Lin CI, 2012 [56]	Retrospective case series	Taiwan	2	NA	Pt1: IVIG + PDN; pt2: 3 MPN pulses + IVIG	1/2 (50%) mortality	Moderate, 3
Aytaç S, 2016 [33]	Retrospective case series	Turkey	6	7%	6/6 GCs (MPN → PDN); 4/6 CsA; 3/6 etoposide; 2/6 IVIG, 2/6 ANK, 2/6 PE (median of 3 sessions)	1/6 (16%) mortality	Moderate, 3
Borgia RE, 2018 [35]	Retrospective cohort	Canada	38	9%	38/38 GCs (26/38 MPN pulses → PDN, 7/38 PDN, 6/38 DEX). 22/38 IVIG; 11/38 CsA, 5/38 etoposide, 2/38 ANK, 2/38 tacrolimus, 1/38 intrathecal MTX, 1/38 alemtuzumab	2/38 (5%) mortality (both refractory cases: both treated with ANK+PE, 1 also received alemtuzumab + intrathecal MTX for severe CNS involvement	High, 3
Buda P, 2018 [57]	Retrospective case series	Poland	1	NA	MPN pulses + CsA	Remission	Low, 3
Sato S, 2022 [58]	Retrospective case series	Japan	11	NA	11/11 GCs (6 MPN pulses); 2/11 IVIG, 2/11 CYC; 4/11 MMF, 1/11 AZA for underlying disease	11/11 remission. 5/6 CNS involvement (1 persistent anxiety disorder)	Moderate, 3
Eloseily EM, 2020 [37]	Retrospective case series	US	5	NA	5/5 ANK. Concomitant treatment reported for a mixed cohort of 8 SLE/MCTD: GCs (87%), CYC (13%)	2/5 died	Moderate, 3
Horne AC, 2021 [27]	Retrospective case series	Sweden	2	NA	2/2 PDN + low dose etoposide	2/2 MAS remission (1 CNS long-term sequelae)	Moderate, 3
Minoia F, 2021 [40]	Retrospective case series	Multinational	2	NA	2/2 MPN pulses, 2/2 CsA, 2/2 CYC, 1/2 IVIG 2/2 PE (1 for TMA, 1 for SLE-MAS severity), 1/2 ECZ (for TMA)	2/2 associated TMA, 2/2 ICU, 2/2 remission (1 severe osteonecrosis, 1 CKD)	High, 3
Rossano M, 2022 [45]	Retrospective case series	Italy	3	NA	3/3 MPN pulses + CsA; 1/3 IVIG	3/3 remission	Moderate, 3

ANK: anakinra; AZA: azathioprine; CKD: chronic kidney disease; CNS: central nervous system; CoE: category of evidence; CsA: ciclosporin A; CYC: cyclophosphamide; DEX: dexamethasone; ECZ: eculizumab; GCs: glucocorticoids; HLH: haemophagocytic lymphohistiocytosis; ICU: intensive care unit; i.v.: intravenous; IVIG: intravenous immunoglobulin; MAS: macrophage activation syndrome; MPN: methylprednisolone; MCTD: mixed connective tissue disease; MMF: mycophenolate mofetil; MTX: methotrexate; NA: not available; PDN: prednisone; PE: plasma exchange; pt: patient; RTX: rituximab; s.c.: subcutaneous; SLE: systemic lupus erythematosus; TMA: thrombotic microangiopathy.

Eleven studies reported detailed information about KD-related MAS in 58 patients (Table 3). Treatment of MAS included GCs (85%), IVIG (73%), CsA (19%) and infliximab (12%). Fifteen patients (26%) received etoposide (11 within HLH protocol). Two KD-MAS patients were successfully treated with IVIG alone [60, 62]. In our SLR, only one patient received anakinra, with rapid remission [29]. Three patients died (5%, all treated with HLH protocol), and only one had persistent coronary artery ectasia.

**Table 3.** Treatment data available on patients with KD-associated MAS.

First author, year (ref)	Type of pubblication	Country	Pts with KD-MAS	MAS prevalence	Treatment	Outcome	Validity score, EULAR CoE
al-Eid W, 2000 [59]	Case report	Saudi Arabia	1	NA	MPN + etoposide	Remission	Low, 4
Latino GA, 2010 [60]	Retrospective case series	Canada	12	1.9%	12/12 IVIG + high dose ASA; 8/12 second and 2/13 third IVIG doses. 11/12 GCs (1 DEX); 3/12 CsA; 1/12 IVIG alone (2 doses)	12/12 remission; 4/12 mild CAA (resolved)	High, 3
Miettunen PM, 2011 [29]	Retrospective case series	Canada	1	NA	MPN, CsA, etoposide → ANK (etoposide discontinued)	Remission	Moderate, 3
Kang HR, 2013 [61]	Retrospective case series	Korea	12	NA	2/12 second IVIG. 10/12 HLH protocol (2 HLH94, 8 HLH2004); 2/12 GC	2/12 died (15%—both received HLH protocol), 1 lost at follow-up, 9/12 remission	Moderate, 3
Wang W, 2015 [62]	Retrospective case series	China	8	1.1%	8/8 IVIG + high-dose ASA; 7/8 GCs (6 MPN, 1 DEX); 1 DEX + etoposide and CsA	1/8 died (13%—received etoposide+CsA); 2/8 CAA (1 persistent); 6/8 discontinued ASA for thrombocytopenia	Moderate, 3
Islam MI, 2017 [54]	Retrospective case series	Bangladesh	1	NA	MPN + oral GCs	NA	Low, 3
Buda P, 2018 [57]	Retrospective case series	Poland	1	NA	MPN + IVIG	Remission	Low, 3
Mousavi MS, 2019 [63]	Retrospective case series	Iran	4	1.8%	4/4 MPN pulses, 1 second IVIG, 2 CsA, 1 IFX, 1 CYC	4/4 remission, no CAA	Low, 4
Pilania RK, 2021 [64]	Retrospective case series	India	12	1.3%	12/12 IVIG + MPN pulses; 1 third IVIG; 4/12 IFX, 1/12 oral CsA	12/12 remission	Moderate, 3
Rivera-Rodriguez L, 2021 [65]	Case report	Mexico	2	NA	2/2 IVIG + MPN; 1 DEX, 1 CsA 2/2 IFX	2/2 remission after IFX	Low, 4
Rhee S, 2022 [66]	Retrospective case series	Korea	4	0.8%	4/4 second IVIG dose; 4/4 additional GCs (1 MPN, 3 DEX); 1 third IVIG, 1HLH-2004, 1 CsA	2/4 ICU. 4/4 remission, no CAAs.	Moderate, 3

ANK: anakinra; ASA: acetylsalicylic acid; CAA: coronary artery aneurism; CoE: category of evidence; CsA: ciclosporin A; CYC: cyclophosphamide; DEX: dexamethasone; GCs: glucocorticoids; HLH: haemophagocytic lymphohistiocytosis; ICU: intensive care unit; IFX: infliximab; IVIG: intravenous immunoglobulin; KD: Kawasaki disease; MAS: macrophage activation syndrome; MPN: methylprednisolone; NA: not available; PDN: prednisone.

### Differences between paediatric sub-specialties and geographic areas

Treatments of the cohort of 362 sJIA-MAS described by Minoia *et al.* [10, 21] were stratified, both according to the geographic area of the referral centre and to the subspecialty of the treating physician. Patients followed in North America more frequently received IVIG and biologics than patients treated in Europe or in other continents (IVIG: North America 54%, Europe 26%, other continents 43%; biologics: North America 34%, Europe 16%, other continents 7%). No significant differences were observed in the percentage of patients treated with GCs, CsA and etoposide. Paediatric haemato-oncologists more frequently used biologic agents (24% *vs* 3%, *P* = 0.02) and etoposide (18% *vs* 10%, *P* = 0.04), whereas paediatric rheumatologists more frequently prescribed CsA (67% *vs* 40%, *P* < 0.0001).

## Discussion

MAS represents a life-threatening condition that requires prompt effective treatment to avoid a potentially fatal outcome; however, the therapeutic approach to MAS is still a challenge for clinicians worldwide. Recently, international collaborative efforts have strived for a common standardized approach [11]. In this context, the METAPHOR project aims to capture the real-life therapeutic strategies in MAS in different clinical settings, and, in particular, the current SLR had the main purpose of uncovering areas in which evidence regarding MAS treatment is still lacking, leading to major discrepancies among practitioners.

Despite the sizable amount of data regarding MAS patients reported in literature, the global level of evidence on treatment outcome is still poor, with a scarcity of comparative data across papers, mainly due to the heterogeneous nature of most studies, the lack of standardized outcome measures, and the high risk of bias in attributing effectiveness or safety to a specific medication or condition. Indeed, outcome data on the concomitant use of different therapies are really difficult to extract, as the timing of start of drugs is rarely specified. Furthermore, although MAS is a unique syndrome, the heterogeneity of the underlying rheumatological backgrounds may differently affect its course and influence the treatment used.

Although not based on any formal clinical trial, high-dose GCs are confirmed as the mainstay of treatment of MAS in all rheumatological backgrounds across the literature, and GCs were used in almost all patients. Together, MPN and prednisolone accounted for 90% of MAS patients, while DEX was mainly used in the context of a HLH protocol and in patients with a potential higher risk of CNS involvement [30]. GCs were mostly used as co-medications, and only 14% of MAS were treated with GCs as monotherapy. Interestingly, these data are in line with what we observed in the cohort of 362 sJIA-MAS, where only 19% of patients survived with GCs alone [21] (unpublished data, courtesy Dr F. Minoia and Dr A. Ravelli). Despite difficulties in assessing their specific efficacy, due to the heterogeneity of conditions reported and co-medications used, the role of GCs in MAS is life-saving especially in low-income countries; of note, a tapering scheme of MPN pulses was proposed for severe MAS in resource limited settings [18]. Furthermore, despite limited numbers, DEX-P was successfully used in MAS refractory to MPN pulses and CsA in Japan [20].

Data on CsA in MAS come only from retrospective cohort studies in which it was mainly used together with several other agents, with variable dosages and routes of administration, making a reliable evaluation of its efficacy highly biased. However, CsA was confirmed as the most frequently used medication besides GCs, with a global positive efficacy and safety profile. CsA is widely accessible at affordable costs and might play a key role in the treatment of MAS refractory to high-dose GCs, especially in low-income countries or in those centres in which biologic medications are not accessible in a timely manner.

Anakinra is by far the most used biologic treatment for MAS, especially for sJIA-MAS. Despite the fact that no (randomized) controlled clinical trial has tested the efficacy of anakinra in MAS, >80% of patients with sJIA-MAS treated with anakinra reported a complete regression of MAS, with a high safety profile. An unbiased evaluation of its efficacy and best therapeutic scheme is impossible to make, given the heterogeneity of the studies included. However, data collected strongly support the use of anakinra in patients with sJIA-associated MAS. Evidence of anakinra’s role in other subtypes of MAS is less robust; however, its safety profile and short half-life make it a valuable option for all sHLH, especially in critical care settings [67]. Data regarding other biologics in MAS are limited. Although no specific biologic used at the indicated regular dose seems to provide full protection against MAS [25, 68, 69], small case-series showed positive results of canakinumab and tocilizumab in sJIA-MAS, raising the possibility of a therapeutic alternative in countries where anakinra is not available; however, further data are needed to confirm this preliminary observation.

Emapalumab is the only medication to be tested in a clinical trial in MAS and showed extremely positive results in high-dose GC-refractory sJIA-MAS with >90% of remission [43]. Given its specific target effect on IFNγ, emapalumab has a highly promising role for all subtypes of MAS, although these preliminary results need to be confirmed in larger cohorts and in patients with other rheumatological backgrounds. Notably, emapalumab is still not accessible in most countries worldwide. Given their effect on the IFNγ pathway, JAK-i could potentially play an important role in MAS treatment; however, so far, evidence on MAS is limited to case reports and to mixed sHLH cohorts. For sJIA-MAS, it should be noted that neither IL-1 nor IL-18 receptors signal through JAKs. IL-18 blockade might also represent a promising approach [70], and an ongoing international trial with a biclonal anti-IL-1β/IL-18 antibody is exploring its effect in monogenic diseases associated with inflammatory MAS (NCT04641442).

Since etoposide is a key medication in HLH protocols, its use in severe MAS was extensively reported, albeit associated with a significant toxicity and mortality. In the 362-cohort of sJIA-MAS described by Minoia *et al.* [21], etoposide was used in almost 12% of cases and was most frequently prescribed by haemato-oncologists [10]. Interestingly, a low-dose etoposide protocol was successfully used in a small cohort of highly refractory MAS patients, with a positive outcome [27], and its role, especially in countries without access to targeted medications, needs to be better explored.

Data reflecting different therapeutic approaches according to geographic areas or sub-specialty of the treating physician were assessable only from one cohort of sJIA-MAS [10, 21]. In a recent survey [71], not included in the SLR due to publication type, GCs were confirmed as the first-line medication for MAS across all the subspecialties; notably, haemato-oncologists preferred DEX over MPN. IL-1 inhibitors were chosen as first-line therapy in MAS more frequently by rheumatologists compared with haemato-oncologists, while etoposide was more frequently the second-line choice of haemato-oncologists.

In conclusion, data regarding MAS treatment are progressively increasing, especially for sJIA-associated MAS, with highly promising results for IL-1 and IFNγ inhibitors. However, the global level of evidence on MAS treatment, especially in other rheumatological conditions, is still poor with high biases and scarce reliability in attributing efficacy to a specific medication, due to the retrospective nature and heterogeneity of most studies and the lack of agreed outcome measures. As a consequence, therapeutic approaches to MAS are still extremely variable, with potential significant discrepancies across different centres and countries. An international effort is needed to optimize therapeutic strategies, reduce gaps in access to medications and harmonize MAS treatment worldwide.

## Supplementary Material

keae391_Supplementary_Data

## Data Availability

All data relevant to the study are included in the article. Data are available upon request from Dr Francesca Minoia (francesca.minoia@policlinico.mi.it).
