# Supplementary material for: Current treatment in macrophage activation syndrome worldwide: a systematic literature review to inform the METAPHOR project
Source: Rheumatology (Oxford). 2024 Jul 26;64(1):32–44. doi: 10.1093/rheumatology/keae391 (PMC11701305; doi:10.1093/rheumatology/keae391)
Supplement: keae391_Supplementary_Data [file keae391_supplementary_data.zip › keae391_Supplementary_Data/rhe-24-0688-File006.docx]

**Supplementary Table S1.** The PICO (Patient-Intervention-Comparison-Outcome) framework adopted to structure the systematic literature review

| **PICOs questions** | |
| --- | --- |
| 1) | Use of glucocorticoids (GCs) in newly diagnosed MAS   1. Favourite type of GC used (MPN vs DEX) 2. Differences in type of GC used due to different rheumatologic underlying disorders 3. Differences in type of GC used due to severity of MAS (i.e. CNS involvement)? |
| 2) | Use of ciclosporin A (CsA) in MAS   1. Frequency of CsA use in MAS 2. Differences in CsA use due to different rheumatologic underlying disorders 3. Favourite route of administration and dosage |
| 3) | Use of anakinra in MAS   1. Frequency of anakinra use in MAS 2. Differences in anakinra use due to different rheumatologic underlying disorders 3. Favourite route of administration and dosage |
| 4) | Use of etoposide in MAS   1. Frequency of etoposide use in MAS 2. Differences in etoposide use due to different rheumatologic underlying disorders 3. Favourite protocol used |
| 5) | Use of JAK-i in MAS   1. Frequency and type of JAK-i used in MAS 2. Differences in JAK use due to different rheumatologic underlying disorders 3. Favourite dosage used |
| 6) | Use of emapalumab in MAS   1. Type of MAS patients treated with emapalumab 2. Favourite dosage |
| 7) | Role of other treatments in MAS (i.e. IVIG, tocilizumab, cyclophosphamide, plasma exchange...) |
| 8) | Role of HSCT in the treatment of MAS |
| 9) | Treatment of MAS complicating rheumatological disorders other than sJIA   1. SLE-MAS 2. KD-MAS |
| 10) | Differences in MAS therapeutic choices across different countries |
| 11) | Role of the subspecialty of the caring physician in MAS therapeutic choices |

CsA: ciclosporin A; CNS: central nervous system; DEX: dexamethasone; GCs: glucocorticoids; IVIG: intravenous immunoglobulin; JAK-i: Janus Kinasis inhibitor; KD: Kawasaki disease; LD: lung disease; MAS: macrophage activation syndrome; MPN: methylprednisolone; sJIA: systemic juvenile idiopathic arthritis; SLE: systemic lupus erythematosus;
