# Supplementary material for: Current treatment in macrophage activation syndrome worldwide: a systematic literature review to inform the METAPHOR project
Source: Rheumatology (Oxford). 2024 Jul 26;64(1):32–44. doi: 10.1093/rheumatology/keae391 (PMC11701305; doi:10.1093/rheumatology/keae391)
Supplement: keae391_Supplementary_Data [file keae391_supplementary_data.zip › keae391_Supplementary_Data/rhe-24-0688-File008.docx]

**Supplementary Data S1. PICO framework and search strings**

**PICO framework**

*Population*: all patients who received a clinical diagnosis of macrophage activation syndrome (MAS) in the context of a known or suspected rheumatologic disorder before 18 years of age.

*Intervention*: each medication used to treat MAS (in particular: glucocorticoids, ciclosporin, etoposide, anakinra, emapalumab, JAK-inhibitors, intravenous immunoglobulins, canakinumab, tocilizumab, plasma exchange)

*Comparator*: for each medication, patients with MAS who did not receive it

*Outcome*: clinical remission as reported by the treating physician, mortality

**Search strings**

**PUBMED**:("lymphohistiocytosis, hemophagocytic"[MeSH Terms] OR "macrophage activation syndrome"[MeSH Terms] OR "hemophagocytic lymphohistiocytos*"[Title/Abstract] OR "haemophagocytic lymphohistiocytos*"[Title/Abstract] OR "reactive hemophagocytic syndrome"[Title/Abstract] OR "macrophage activation syndrome"[Title/Abstract] OR "HLH"[Title/Abstract] OR "SHLH"[Title/Abstract] OR "secondary hemophagocytic lymphohistiocytos*"[Title/Abstract] OR "secondary haemophagocytic lymphohistiocytos*"[Title/Abstract]) AND ("treat*"[Title/Abstract] OR "manag*"[Title/Abstract] OR "therap*"[Title/Abstract]) AND 1997/01/01:2022/12/31[Date - Publication] AND "english"[Language]

**EMBASE**: ('hemophagocytic syndrome'/exp OR 'macrophage activation syndrome'/exp OR ‘hemophagocytic lymphohistiocytos*’:ti,ab,kw OR ‘haemophagocytic lymphohistiocytos*’:ti,ab,kw OR ‘reactive hemophagocytic syndrome’:ti,ab,kw) AND (‘treat*’:ti,ab,kw OR ‘manag*’:ti,ab,kw OR ‘therap*’:ti,ab,kw) AND [embase]/lim AND ('article'/it OR 'article in press'/it) AND (1997:py OR 1998:py OR 1999:py OR 2000:py OR 2001:py OR 2002:py OR 2003:py OR 2004:py OR 2005:py OR 2006:py OR 2007:py OR 2008:py OR 2009:py OR 2010:py OR 2011:py OR 2012:py OR 2013:py OR 2014:py OR 2015:py OR 2016:py OR 2017:py OR 2018:py OR 2019:py OR 2020:py OR 2021:py OR 2022:py) AND [english]/lim
