# Supplementary material for: Current treatment in macrophage activation syndrome worldwide: a systematic literature review to inform the METAPHOR project
Source: Rheumatology (Oxford). 2024 Jul 26;64(1):32–44. doi: 10.1093/rheumatology/keae391 (PMC11701305; doi:10.1093/rheumatology/keae391)
Supplement: keae391_Supplementary_Data [file keae391_supplementary_data.zip › keae391_Supplementary_Data/Supplementary Table 2_ref edited.docx]

**Supplementary Table 2.**  Detailed information on the papers finally included in the systematic literature review

| **First author, year (ref)** | **Journal** | **Study type** | **MAS patients reported** | **Country** | **Treatments reported** | **Outcome** | **Validity score,**  **EULAR CoE** |
| --- | --- | --- | --- | --- | --- | --- | --- |
| Grom AA, 2016 (25) | Arthritis Rheumatol | Multicentre perspective cohort | 21 probable sJIA-MAS, 10 possible sJIA-MAS | US | 21 probable MAS, 19 in the CNK arm: 6 GCs alone, 3 GCs + IVIG, 5 GCs + CsA, 3 GCs + CSA+IVIG, 2 GCs + CSA + VP16  10 possible MAS: GCs alone | 3 patients died: 2 in the CNK arm (GCs+CsA+IVG and GCs+CsA+VP16) and 1 in the placebo group (GCs +IVIG). 1 patient had PRES post CsA and bone marrow suppression after etoposide. | High, 2B |
| Wang W, 2015 (62) | Semin Arthritis Rheum | Single-centre retrospective case series | 8 KD-MAS | China | 8/8 IVIG + high-dose ASA; 7/8 GC (6 MPN, 1 DEX); 1 DEX + etoposide and CsA | 1/8 died (13% - received etoposide+CsA); 2/8 CAA (1 persistent); 6/8 discontinued ASA for thrombocytopenia | Moderate, 3 |
| Minoia F, 2014 (21) | Arthritis Rheumatol | Multicentre retrospective case series | 362 sJIA-MAS | Multinational | GCs 98% (iv 89%), CsA 61%, IVIG 36%, etoposide 12% (prescribed more frequently by hemato-oncologists [18%], than by rheumatologists [10%]), anakinra 10% (94% MAS onset after 2006). | 35% ICU admission  Mortality 8% | High, 3 |
| Kang HR, 2013 (61) | Blood Res | Multicentre retrospective case series | 12 KD-MAS | Korea | 2/12 2^nd^ IVIG. 10/12 HLH protocol (2 HLH94, 8 HLH2004); 2/12 GCs | 2/12 died (15% - both due to infections during HLH protocol) – 1 lost at follow-up) 9/12 remission  Overall survival rate at 4 years 81% with a median follow up of 45 months | Moderate, 3 |
| Bennett TD, 2012 (30) | Arthritis Rheum | Multicentre retrospective case series | 102 JIA-MAS, 19 SLE-MAS | US | GC 93% (MPN 83%; DEX was given more frequently to SLE [32%] than sJIA [14%] p = 0.05) SLE received more CYC (21% vs 3%, p = 0.01) and MMF (32% vs 2%, p < 0.001) than sJIA.  Only sJIA (15 patients) received ANK: 14/15 GC, 5/15 CsA, and 1/15 VP16; 11/15 received ANK within D3 and 13/15 within D6.  CsA and IVIG did not differ between SLE and sJIA (47% vs 44% and 37 vs 18%, respectively).  PE used in 2 patients with SLE  2 sJIA patients received HSCT | ICU admission 33%  Mortality 7% (SLE 11% vs JIA 6, p 0.6) SLE had higher ICU admission (63% vs 27%, p = 0.002), longer hospitalization (median 25 days vs 6 days, p < 0.001), and higher costs (median $126,600 versus $16,886, p < 0.001) JIA. | Moderate, 3 |
| Lin CI, 2012 (56) | Clin Rheumatol | Single-centre retrospective case series | 8 MAS (4 sJIA, 2 SLE, 1 MCTD, 1 APS) | Taiwan | 8/8 GC (at least 1-2 mg/kg/day PDN equivalent)  3/8 (sJIA) GC alone with positive outcome  6/8 IVIG 1^st^ line treatment (1 g/kg/day for 2 days): 2/6 response (33%), 3/4 who failed IVIG were given MPN pulses (2/3 remission).  2/8 (sJIA) CsA with positive outcome  1/8 (sJIA) was given ETA without response (🡪 MPN pulses + CsA) | 8/8 ICU admission  Complications reported: 2/8 shock 2/8 opportunistic infection (PCP), 2/8 MOF, 1/8 myopathy (Patient 4).  Mortality 12.5% (1/8, seen before 2000) | Moderate, 3 |
| Latino GA, 2010 (60) | J Pediatr Hematol Oncol | Single-centre retrospective case sesiries | 12 KD-MAS | Canada | 12/12 IVIG + high dose ASA; 8/12 2^nd^ and 2/13 3^rd^ IVIG doses. 1/12 IVIG alone (2 doses)  11/12 GC (1 DEX); 3/12 CsA  Median treatment duration: 7 weeks (range 1-33 weeks) | 12/12 remission; 6/12 acute cardiac complications (heart failure and arrhythmias)  4/12 mild CAA (resolved) | High, 3 |
| Nakakura H, 2009 (72) | Ther Apheresis Dial | Case report | 1 sJIA-MAS | Japan | MPN pulses (30 mg/kg/day x 3 days) + CsA iv (1 mg/kg/day), without improvement and CMV infection.  After CMV treatment, iv DEX (10 mg/m^2^) + CsA iv (1.5 mg/kg/day) for 1 week 🡪 MPN pulses (10-20 mg/kg/day for 5 days)  Due to persistent cytopenia PE was started using FFP with improvement after 3 sessions | CMV concomitant infection  Complete recovery | Low, 4 |
| Zeng HS, 2008 (73) | World J Pediatr | Single-centre retrospective case series | 13 sJIA-MAS | China | 13/13 DEX (6/13 DEX alone, 3/13 DEX + VCR, 2/13 DEX + VP16, 1 DEX + VCR + etoposide, 1 DEX + CsA + IVIG + etoposide)  1/13 PE | Mortality 3/13 (23%)  10/13 patients recovered completely | Low, 3 |
| Lambotte O, 2006 (53) | Medicine | Single-centre retrospective case series | 12 SLE-MAS (15 episodes) | France | 14/15 GCs (9 MPN + PDN, 3 PDN); 2/15 oral PDN in monotherapy;  6/15 IVIG (5/6 as 1^st^ line, 3/6 1^st^ line monotherapy); 2/15 CYC (1 after failure of VP16 + CsA and RTX)  1 pt without specific treatment | Patient without specific treatment relapsed 🡪 MPN;  3/3 IVIG monotherapy did not respond 🡪 GCs  5/15 ICU | Moderate, 3 |
| Kounami S, 2005 (23) | Acta Haematol | Single-centre retrospective case series | 5 sJIA-MAS (9 episodes) | Japan | 6/9 high doses GCs; 5/9 CsA  3 episodes successfully treated with CsA (continuous iv 3 mg/kg/day), as 1^st^ line without increasing GC. | Mortality 11% (1 patient died, treated with MPN pulses + IVIG + etoposide + PE) | Low, 3 |
| Al-Eid W, 2000 (59) | Pediatr Hematol Oncol | Case report | 1 KD-MAS | Saudi Arabia | MPN pulses + etoposide + G-CSF | Complete recovery | Low, 4 |
| Charlesworth JEG, 2021 (38) | Pediatr Blood Cancer. | Single-centre retrospective case series | 3 sHLH (1 excluded as malignancy-associated) | UK | 2/2 received continuous anakinra iv infusion (Pt1: 12 mg/kg/day 🡪 48 mg/kg/day; Pt2: 11 mg/kg/day)  Pt1: etoposide + CsA | 2/2 remission | High, 4 |
| Loganathan S, 2021 (18) | Indian J Pediatr | Case report | 2 sJIA-MAS | India | 2/2 MPN pulses (30 mg/kg/day) for 5-10 days, followed by 20 mg/kg/day for 3 days, 15 mg/kg/days for 3 days, 10 mg/kg/days for 3 days  Pt1: etopside + anakinra on D27 (MAS already in remission)  Pt2: CsA | 2/2 remission | Low, 3 |
| Nakagishi Y, 2016 (19) | Mod Rheumatol | Multicentre retrospective case series | 4 sJIA-MAS | Japan | 4/4 treated with DEX-P  Pt1 (SLE) and Pt2 (sJIA) refractory to MPN pulses (1 g/day for 3 days) + iv CsA (1 mg/kg/day).  Pt 3 and 4 (sJIA) treated 1^st^ line with DEX-P + iv CsA (1 mg/kg/day continuous) | 4/4 remission | Low, 3 |
| Ozturk K, 2015 (31) | Rheumatol Int | Case report | 1 sJIA-MAS | Turkey | MAS refractory to high-dose GC (MPN pulses [1 g/day for 3 days on w1 and w3] and DEX on w2] + IVIG + etoposide (150 mg/m^2^ 3 times on w2) + TAC (already ongoing for previous liver transplant) switched to CsA (5 mg/kg/day) on w3 (TAC toxicity).  ATG (1.5mg/kg/day for 6 doses) started on w5 + anakinra (2 mg/kg/day) from w6 (and continued as maintenance therapy) | Complete recovery | Low, 4 |
| Sato S, 2022 (58) | Ann Rheum Dis | Case report | 1 sJIA-MAS with lung disease | Japan | MAS refractory to MPN pulses + IVIG + CsA, with associated LD  PE (8 sessions) with progressive improvement | Complete recovery | Moderate, 4 |
| Miettunen PM, 2011 (29) | Rheumatology | Multicenter retrospective case series | 12 MAS (8sJIA, 2 AAV, 1 KD, 1 ARF) | Canada, US | 12/12 refractory to MPN (100%), 9/12 IVIG (as 2^nd^ line), 10/12 CsA (2 as 2^nd^ line and 8 as 3^rd^ line after IVIG), 2/12 VP16, 1/9 ETA  12/12 treated with ANK (2 mg/kg/day sc, max 100/mg/day); etoposide, anti TNF stopped once anakinra started; all other treatments continued | 12/12 response to anakinra (median time to remission: 13 days)  5/12 ICU admission | Moderate, 3 |
| Cortis E, 2006 (52) | Acta Paediatr Suppl | Single-centre retrospective case series | 7 MAS (6 sJIA and 1 SLE) | Italy | 7/7 MPN pulses + CsA  1/7 MAS relapse refractory to MPN and CsA 🡪 ETA (0.4 mg/kg twice a week) | 7/7 response to MPN + CsA  1/7 relapsed and had a positive response to ETA | Low, 3 |
| Islam MI, 2017 (54) | Mymensingh Med J | Single-centre retrospective case series | 10 MAS (7 sJIA, 2 SLE, 1 KD) | Bangladesh | 10/10 MPN pulses (30 mg/kg/day for 3 days followed by oral PDN (2-3mg/kg/day).  2/10 oral CsA (3mg/kg/day). | Mortality 30% (3/10; coagulopathy and sepsis)  7/10 complete recovery: mean time to clinical recovery 10.5 days; mean time to hematological and biochemical normalization 19.3 and 26.5 days | Low, 3 |
| Wu J, 2021 (46) | Mod Rheumatol | Multicentre retrospective case series | 14 sJIA-MAS | China | 14/14 refractory to MPN pulses (15–30 mg/kg/day for 3 days) followed by PSL (2 mg/kg/day, max 60 mg/day); 12/14 CsA, 11/14 IVIG (2 g/kg/dose)  14/14 treated with TCZ (12 mg/kg/day < 30 kg and 8 mg/kg/day > 30 kg every 2 weeks iv) | 1 patient lost at follow up  13/14 response to TCZ  No adverse event reported, no death | Moderate, 3 |
| Sawhney S, 2001 (74) | Arch Dis Child | Single-centre retrospective case series | 9 MAS (7 sJIA MAS,1 ERA, 1 CINCA) | UK | 9/9 GC (8 MPN pulses, 1 oral PDN)  6/9 CsA (2–5 mg/kg/day orally, 1 combined to VP16); 2/9 CYC (1 for suspected PAN and 1 with severe renal impairment) | 2/9 died (sJIA)  1/9 MAS relapse 1 month after (infection trigger) | Low, 3 |
| Mouy R, 1996 (26) | J Pediatr Hematol Oncol | Single-centre retrospective case series | 5 sJIA-MAS (9 episodes) | France | 5/5 treated with CsA (3 patients refractory to high dose MPN; in 2 patients CsA was successfully used as 1^st^ line without modification of GC treatment)  In 7/9 episodes CsA was given iv (2-5mg/kg/day) initially, then switched orally (1-4mg/kg/day); 2/9 episodes were initially treated with CsA orally (4-8 mg/kg/day) | 5/5 response to CsA with apyrexia within 24-48 hours.  Time to biochemical normalization up to 5 weeks | Moderate, 3 |
| Stephan JL, 2001 (22) | Rheumatology | Multicentre retrospective case series | 24 MAS (18 sJIA, 2 SLE, 2 poly-JIA, 2 undefined); 29 episodes | France | 21/29 episodes iv GC (0.75 mg/kg/day – 30 mg/kg/day pulses)  4/29 received IVIG as 1^st^ line treatment without improvement  7 patients received CsA (iv 3-7 mg/kg/day in 4 pts, orally in 2 pts)  1 patient received VP16 | 2/24 died (1 cardiac shock treated with IVIG and GC; 1 due to VZV infection after GC)  Adverse events reported: mild hypertension (1mild and reversible renal impairment (1)  22/24 patients achieved complete response after GC +/- CsA | Moderate, 3 |
| Baglan E, 2022 (42) | Aktuel Rheumatol | Single centre retrospective cohort | 10 sJIA MAS | Turkey | 10/10 high dose MPN, 8/10 PE + IVIG  5/10 anakinra, 1 anti IL-6.  1 patient received CsA without improvement | 9/10 complete recovery, 1 patient with recurrent MAS | Moderate, 3 |
| Palmblad K, 2021 (28) | Mol Med | Single centre retrospective case series | 3 sJIA-MAS, 1 SLE-MAS | Sweden | 4/4 refractory to MPN pulses, 3/4 CsA (2 sJIA and 1 SLE), 1/4 (sJIA) ANK (4 mg/kg/day), 2/4 (sJIA) to TCZ, 1 (SLE) PE.  4/4 treated with low dose etoposide (50-100 mg/m^2^/week) due to severe CNS involvement (3) or refractory cytopenia (1) | 4/4 complete recevery | Low, 3 |
| Phadke O, 2021 (39) | Pediatr Rheumatol | Single centre retrospective case series | 14 MAS (10 sJIA, 3 SLE, 1 vasculitis) | US | 14/14 treated with ANK iv  ANK starting dose: 1.7 - 10 mg/kg/day iv  Maximum dose: 4.2–15.4 mg/kg/day iv  (max 400 mg/day)  Iv ANK protocol: sc formulation mixed in normal saline with (1 ml of normal saline: 1 mg of anakinra), administered iv over 30 minutes  Median duration of ANK treatment: 10 days  Maximum duration of ANK treatment: 85 days | 2/10 died (1 sJIA-MAS due to sepsis after MPN, DXA, etoposide, JAK-i; 1 vasculitis-MAS with stroke and MOF after CYC, RTX, ECZ)  No SAE reported  1/10 elevation of liver enzymes on ANK 8 mg/kg/day (reversed with ANK discontinuation)  8/10 complete recovery | Moderate, 3 |
| Horne A, 2021 (27) | J Rheumatol | Single centre retrospective case series | 7 MAS (5 sJIA, 2 SLE) | Sweden | 7/7 refractory to MPN pulses, 3/7 ANK (2.7-15mg/kg/day), 3/7 CsA  7/7 successfully treated with low-dose VP16 (50–150 mg/m^2^/week)  1 patient (sJIA) received also RTX due to severe EBV infection, 2 patients (sJIA) received IVIG and 1 patient (SLE) received PE | 7/7 positive response to low-dose VP16  Adverse events reported: 2 severe neutropenia (1 sepsis); 1 patient (SLE) severe CNS sequelae | Moderate, 3 |
| Minoia F, 2021 (40) | J Pediatr | Multicentre retrospective case series | 23 MAS with TMA (17 sJIA, 6 SLE, 1 JDM, 1 MCTD, and 2 UCTD) | Multinational | 23/23 high dose GC  MAS treatment: 17/23 IVIG, 14/23 CsA, 10/23 ANK, 4/23 etoposide, 4/23 CYC, 1/23 MMF  Treatment of TMA: 17/23 PE, 9/23 ECZ, 6/23 RTX | 20/23 ICU admission  7/20 long term sequelae  3/23 died | High, 3 |
| Pilania RK, 2021 (64) | Rheumatology | Single-centre case series | 12 KD-MAS | India | 12/12 IVIG + MPN pulses  4/12 IFX (5–10 mg kg single dose), 1/12 oral CsA (3– 5 mg/kg/day) 1 patient received 3 IVIG doses | 12/12 complete recovery | Moderate, 3 |
| Yu TY, 2021 (24) | J Formos Med Assoc | Single-centre case series | 7 MAS (3 SLE, 2 sJIA, 1 KD, 1 Kikuchi) | Taiwan | 6/7 GC, 6/7 IVIG, 2/7 CsA, 3/7 etoposide, 1 HSCT | 2/7 died  5/7 complete recovery | Moderate, 3 |
| Rivera Rodriguez L, 2021 (65) | J Pediatr Hematol Oncol | Case report | 2 KD-MAS | Mexico | 2/2 treated with IFX (5 mg/kg/dose)  Pt1 refractory to IVIG (2 doses), MPN pulses + CsA  Pt2 refractory to IVIG (2 doses), MPN pulses and DEX | 2/2 complete recovery after IFX | Low, 4 |
| Aydin F, 2021 (41) | Turk J Ped Dis | Single-centre retrospective case series | 7 sJIA MAS | Turkey | 7/7 GC, 5/7 CsA, 4/7 ANK (in 3 of them switched to CNK), 1/7 IVIG | 1/7 died (GC, ANK 🡪 CNK)  6/7 complete recovery | Low, 3 |
| Pal P, 2020 (75) | Int J Rheum Dis | Single-centre retrospective case series | 31 MAS (26 sJIA, 4 SLE, 1 KD) | India | 31/31 GC; 15/31 CsA; 9/31 IVIG (5 as 1^st^ line, 4 as 2^nd^ line)  12 patients were treated with MPN+CsA, 10 with MPN alone, 6 with MPN+IVIG and 3 with IVMP+CsA+IVIG  2 patients refractory to MPN+CsA+IVIG received HLH04 protocol (2/2 died) | 10/31 died (mortality 32%) | Low, 3 |
| Zou LX, 2020 (47) | World J Pediatr | Single-centre retrospective case series | 80 MAS (53 sJIA, 10 KD, 9 SLE, 5 JDM, 2 MCTD, 1 AAV) | China | 79/80 GC (80% MPN pulses 10-30 mg/kg/day for 3–5 days, followed by oral PSL 1–2 mg/kg/day).  44/80 IVIG (26 sJIA, 10 KD, 8 CTD)  43/80 CsA (31 sJIA, 10 CTD, 2 KD)  10/80 (sJIA) received biologic treatment (8 TCZ, 2 IFX); 1 sJIA was treated with TCZ alone  6/80 PE (5 sJIA, 1 SLE)  1 HSCT | 6/80 died (6 sJIA due to MOF, 1 JDM; due to fungal infection; mortality 7.5%) | Moderate, 3 |
| Eloseily EM, 2020 (37) | Arthritis Rheum | Single-centre retrospective case series | 28 MAS (13 sJIA, 5 SLE, 3 MCTD, 7 others) | US | 28/28 treated with ANK (sJIA: 2.9 - 11.9 mg/kg/day; SLE/MCTD: 2-48mg/kg/day [latter as continuous IV infusion])  Concomitant treatments:  sJIA: GCs (54%), CsA (23%)  SLE: GCs (87%), CYC (13%) | 13/13 sJIA-MAS complete recovery  2/5 SLE died (1 due to sepsis treated with MPN, ANK and CYC; 1 due to MOF treated with MPN and ANK)  Parameters associated with lower mortality: sJIA, early introduction of ANK (≤5 days of hospitalization).  Thrombocytopenia was associated with higher mortality | Moderate, 3 |
| Mousavi MS, 2019 (63) | Iran J Pediatr | Single-centre retrospective case series | 4 KD-MAS | Iran | Pt1: MPN iv, CYC, CsA (orally 5mg/kg/day).  Pt2: MPN pulses (30 mg/kg/day for 3 days) + IFX (5 mg/kg/dose)  Pt3: MPN pulses (30 mg/kg/day for 3 days) + IVIG (2 g/kg)  Pt4: MPN pulses (30 mg/kg/day for 3 days) | 4/4 complete recovery  No cardiac sequelae | Low, 4 |
| Sonmez HE, 2018 (36) | Clin Rheumatol | Single-centre retrospective case series | 15 sJIA, 2 AID (1 CINCA, 1 MKD)  19 MAS episodes | Turkey | 19/19 MPN pulses (30 mg/kg/day for 3 days followed by oral PSL).  19/19 treated with ANK as 1^st^ line together with MPN; starting dose 2 mg/kg/day, increased up to 4-6 mg/kg/day  Median time to ANK start: 1 day after hospitalization  Concomitant treatment reported:  12/19 CsA, 6/19 PE for refractory thrombocytopenia, 3/19 etoposide (1 single dose)  IVIG were used as addition therapy (% not reported) | 13/15 sJIA complete recovery  2/15 sJIA recurrent MAS  No SAE reported  1 patient developed vitiligo and was switched to CNK | Moderate, 3 |
| Buda P, 2018 (57) | Arch Med Sci | Single-centre retrospective case series | 24 MAS (12 sJIA, 1 SLE, 1 KD, 10 other) | Poland | sJIA: 12/12 GC (6 MPN pulses 30 mg/kg/day as 1^st^ line, 6 PDN 1-2mg/kg/day as 1^st^ line 🡪 3 required MPN pulses); 9/12 CsA, 7/12 IVIG, 2/12 IFX (1 received also etoposide+PE)  SLE: MPN pulses 30 mg/kg/day + CsA  KD: MPN + IVIG | 24/24 complete recovery | Low, 3 |
| Aytac S, 2016 (33) | Rheumatol Int | Single-centre retrospective case series | 34 MAS (28 sJIA with 31 episodes, 6 SLE)  37 MAS episodes | Turkey | 37/37 high dose GC (MPN pulses 30 mg/kg/day 3–5 days followed by oral PSL 1–2 mg/kg/day)  sJIA: 23/31 CsA, 21/31 IVIG, 13/31 ANK (all patients diagnosed after 2011 received ANK), 13/31 PE, 10/31 VP16  SLE: 4/6 CsA, 2/6 IVIG, 3/6 etoposide 2/6 ANK, 2/6 PE | 4/34 died (3 sJIA and 1 SLE, mortality 12%)  Parameters associated with exitus: longer time to hospital admission (16.5 days vs 7 days p 0.046), hepatosplenomegaly (100% vs 21%, p 0.005), PE (100% vs 33%, p 0.021) | Moderate, 3 |
| Gupta AA, 2009 (51) | J Pediatr Hematol Oncol | Single-centre retrospective case series | 14 MAS | Canada | 5/14 GC, 5/14 IVIG, 2/14 HLH protocol, 2/14 CsA | 1/14 died (SLE treated with HLH protocol) | Moderate, 3 |
| Minoia F, 2015 (10) | J Rheumatol | Multicentre retrospective case series | 362 sJIA-MAS | Multinational | For patient cohort treatment details please see ref 20. Herein, a stratification of MAS treatment according to geographic location, specialty of the caring physician and presence of hemophagocytosis and outcome was reported.  Geographic location: patients followed in North America received more frequently IVIG and biologics than patients treated in Europe or in other continents (54% vs 26% and 43%, 34% vs 16% and 7%)  Specialty of caring physician: hematologists used more frequently biologics (24% vs 3%, p = 0.02) and etoposide (18% vs 10%, p = 0.04), while rheumatologists ciclosporin (67% vs 40%, p < 0.0001)  Patients with hemophagocytosis received more cyclosporine (74.4% vs 50.0%, p < 0.0001), IVIG (44.9% vs 29.4%, p = 0.003), and etoposide (18.8% vs 6.0%, p = 0.0003).  Patients with severe outcome (ICU, death) were more commonly given cyclosporine, IVIG, and etoposide. | See ref 20 Outcome was overall worse in patients followed in other continents than Europe and North America. | High, 3 |
| Singh S, 2012 (76) | Rheumatol. Int | Single-centre retrospective case series | 6 sJIA-MAS | India | 6/6 GCs (4 MPN pulses, 2 oral PSL 2 mg/kg/day)  2/6 IVIG (MPN) | 1/6 died (treated with MPN and IVIG) | Low, 3 |
| Gokce M, 2012 (55) | Lupus | Single-centre retrospective case series | 6 SLE-MAS | Turkey | 6/6 GCs (3 MPN, 3 DEX); 3/6 HLH-2004 protocol; 3/6 CsA + IVIG;  2/6 PE (TMA) | 1/6 died due to secondary infections and MOF treated with HLH-2004 protocol despite aggressive treatment. | Low, 3 |
| Sato S, 2019 (58) | Rheumatol Adv Pract | Single-centre retrospective case series | 11 SLE-MAS | Japan | 11/11 GCs (6 MPN pulses); 2/11 IVIG, 2/11 CYC; 4/11 MMF, 1/11 AZA for underlying disease | 11/11 complete MAS recovery.  5/6 CNS involvement (1 persistent anxiety disorder) | Moderate, 3 |
| Barut K, 2015 (32) | Turk Pediatri Ars | Single-centre retrospective case series | 10 sJIA-MAS | Turkey | 10/10 GCs, 8/10 CsA, 5/10 ANK, 4/10 CNK, 2/10 PE | 9/10 complete recovery  1/10 recurrent MAS | Low, 3 |
| Silva MF, 2018 (34) | Blood Adv | Multicentre retrospective case series | 4 sJIA-MAS | UK | 4/4 received HSCT for refractory sJIA-MAS  Conditioning regimen was Fludarabine, Melphalan Alemtuzumab for all patients.  Previous treatments: 4/4 high dose GC, 4/4 CsA, 3/4 ANK, 1/4 etoposide, ATG, TCZ, IFX each | 1/4 died  3/4 complete MAS recovery  SAE: 2/4 GVHD, 4/4 severe infections (CMV, EBV, adenovirus, fungal infection), 1 severe neutropenia, 1 AVN | Moderate, 3 |
| Sahu SK, 2020 (77) | Int. J. Res. Pharm. Sci. | Single-centre retrospective case series | 3 sJIA-MAS | India | 3/3 GC + CsA, 1/3 IVIG | 3/3 complete recovery  2/3 ICU admission | Moderate, 4 |
| Borgia RE, 2018 (35) | Arthritis Rheumatol | Singlecentre retrospective cohort | 38 SLE-MAS | Canada | 38/38 GCs (26/38 MPN pulses 🡪 PDN, 7/38 PDN, 6/38 DEX). 22/38 IVIG; 11/38 CsA, 5/38 etoposide, 2/38 ANK, 2/38 tacrolimus, 1/38 intrathecal MTX, 1/38 alemtuzumab | 2/38 died (both refractory cases: both treated with ANK+PE, 1 also received alemtuzumab + intrathecal MTX for severe CNS involvement) | High, 3 |
| He T, 2023 (50) | Front Pediatr | Single-centre retrospective case series | 3 sJIA-MAS | China | 3/3 refractory to high doses GCs (2 MPN pulses, 1 DEX) + TCZ; 1/3 also etoposide (none treated with anti IL-1 or CsA)  2/3 received ruxolitinib (2.5 mg x2/day 🡪 5 mg/day); 1 patient received tofacitinib (5 mg/day) without response and was switched to ruxolitinib (7.5 mg x 2/day) | 3/3 complete MAS recovery but required association with CNK to control sJIA  No adverse event | Moderate, 4 |
| Lukjanoviˇca K, 2023 (48) | Medicina | Single-centre retrospective case series | 10 sJIA-MAS | Latvia | 10/10 MPN + CsA 🡪 8/10 were added TCZ with positive response | 10/10 complete recovery  8/8 treated with TCZ had positive response within 48 hours  Adverse events: 1 CsA-induced PRES  No serious complications were associated with the use of TCZ (1 mild persistent thrombocytopenia) | Moderate, 3 |
| De Benedetti F, 2023 (43) | Ann Rheum Dis | Single-arm, open label, multicentre clinical trial | 14 sJIA-MAS | Multinational | 14/14 refractory to high-dose GCs (8/14 also to CsA and 7/14 also to ANK up to 15 mg/kg/day)  14/14 treated with emapalumab  Emapalumab protocol: 6 mg/kg on D0, followed by 3 mg/kg every 3 days until D15, then twice weekly until D28 (all patients received at least 3 administration); frequency or dose could be increased or treatment prolonged if required  Median treatment duration: 27 days (range, 7–39)  Concomitant treatments: GC, CsA (discontinued in 2 patients within D10, and in further 4 during follow up), ANK (continued in 4 patients at ≤4 mg/kg and in one patient at 7.5 mg/kg).  14/14: acyclovir prophylaxis | At 8 weeks, 13/14 met MAS remission criteria (93% response)  1/14 never met remission criteria only due to LDH levels 1.5-fold above the ULN (emapalumab stopped after 3 administrations due to clinical remission).  Median time to MAS remission: 25 days (the earliest 9 days)  Median daily dose of PDN-equivalent at w8: 0.56 mg/kg/day  No deaths. 1 SAE (CMV infection, treated with standard care).  Most frequently reported adverse events were viral infections (2) and positive tests for viral infectious agents (4) in the absence of clinical symptoms (mainly CMV). Rate of adverse events and infections not increased during concomitant treatment with ANK compared with EMP alone  6/14 had a flare of sJIA (6/9 patients not treated with ANK together with EMP). No sJIA flares in the 5 patients who continued ANK together with emapalumab | High, 2A |
| Shimizu M, 2023 (20) | Int J Rheum Dis. | Multicentre retrospective case series | 28 sJIA-MAS | Japan | 9/28 treatment naïve, 8/28 on GC, 11/28 on TCZ  1^st^ line: 28/28 GCs (15 DEX-P, 7 PSL, 6 MPN) + 14/28 CsA  2^nd^ line: 1MPN, 5 DEX-P, 5 CsA, 2 PE  3^rd^ line: 2 PE  DEX-P was given iv at 3.2–8.6 mg/m^2^/day (max 10 mg/d)  CsA was given iv by continuous infusion (0.83–3.3 mg/kg/day) in 11 patients and orally (2.7–5.7 mg/kg/day) in 5 patients | 28/28 complete recovery.  No SAE related to DEX-P | Moderate, 3 |
| Chellapandian D, 2023 (44) | Front in Pediatr | Case report | 1 sJIA-MAS | US | 4-year-old girl with sJIA complicated by recurrent MAS (1^st^episode at 21 m, treated with MPN pulses, ANK; 2^nd^ at 30 m, treated with MPN pulses, ANK escalation to 3 mg/kg/day, 3^rd^ at 36 m, treated with MPN pulses, ANK 4 mg/kg/day) and progressive LD.  Due to refractory MAS, emapalumab was started (1^st^ dose 6 mg/kg, then 3 mg/kg twice weekly for 4 weeks) + oral PDN 0.5-1 mg/kg/day 🡪 MAS remission  The patient received a matched sibling donor allo-HSCT after a reduced-intensity conditioning regimen with fludarabine/melphalan/thiotepa and alemtuzumab, along with TAC and MMF for GVHD prophylaxis. | At 20 months follow-up: full donor engraftment with complete donor-derived immune reconstitution + complete resolution of sJIA and marked improvement in  LD (normalization of serum interleukin-18 and CXCL9 levels) | High, 4 |
| Rhee S, 2023 (66) | Children | Single-centre retrospective case series | 4 KD-MAS | Korea | 4/4 2^nd^ IVIG dose; 4/4 additional GC (1 MPN, 3 DEX); 1 3^rd^ IVIG, 1 HLH-2004, 1 CsA | 2/4 ICU admission.  4/4 complete recovery, no cardiac sequelae | Moderate, 3 |
| Kostik MM, 2022 (49) | Front in Pediatr | Single-centre retrospective case series | 8 sJIA-MAS | Russian Federation | 8/8 MAS refractory to high-dose MPN, 5/8 IVIG, 3/8 CsA.  5/8 already on CNK and 3/8 on TCZ  8/8 treated with CNK for MAS  CNK range 2-12 mg/kg/dose  In 3 patients CNK was used as 1^st^ line biologic treatment (4 mg/kg/day).  3 patients developed MAS under CNK standard treatment and responded to an escalation of CNK up to 12 mg/kg/day | 7/8 complete recovery  1 patient required the addition of tofacitinib to control recurrent MAS  2 patients with sJIA-LD: 1 switched to TCZ for persistent arthritis after 3 years from MAS; 1 maintained on CNK together with MMF with stable lung disease control | Moderate, 3 |
| Rossano M, 2023 (45) | Children | Single-centre retrospective case series | 12 MAS (6 sJIA, 3 SLE, 2 JDM, 3 undefined) | Italy | 12/12 MPN pulses (10–30 mg/kg/day for 3-5 days) + CsA; 4/12 IVIG, 3/12 ANK (5 mg/kg/day sc) | 11/12 complete response  1/12 developed CNS sequalae (sJIA, with a triggering sepsis by Staphylococcus and brain hemorrhage before MAS diagnosis) | Moderate, 3 |

AAV: ANCA (anti neutrophil cytoplasmic antibodies) associated vasculitis; AID: autoinflammatory disease; ANK: anakinra; APS: antiphospholipid syndrome; ARF: acute rheumatic fever; ASA: acetylsalicylic acid; ATG: anti-thymocyte globulin; AZA: azathioprine; AVN: avascular necrosis; CAA: coronary artery aneurism; CINCA: cryopyrin associated periodic syndrome; CNK: canakinumab; CoE: category of evidence; CMV: Cytomegalovirus; CNS: central nervous system; CsA: ciclosporin A; CYC: cyclophosphamide; DEX: dexamethasone; DEX-P: dexamethasone palmitate; EBV: *Ebstein-Barr* virus ECZ: eculizumab; ETA: etanercept; ERA: enthesitis related arthritis; GCs: glucocorticoids; G-CSF: granulocyte colony stimulating factor; GVHD: graft versus host disease; i.v. intravenous; HLH: hemophagocytic lymphohistiocytosis; HSCT: hematopoietic stem cell transplant; ICU: intensive care unit; IFX: infliximab; IVIG: intravenous immunoglobulin; JAK-i: Janus Kinasis inhibitor; JDM: juvenile dermatomyositis; KD: Kawasaki disease; LD: lung disease; MAS: macrophage activation syndrome; MMF: mycophenolate mofetil; MPN: methylprednisolone; MCTD: mixed connective tissue disease; MOF: multiorgan failure; MTX: methotrexate; MKD: mevalonato kinase deficiency; NA: not available; PAN: panarteritis nodosa; PCP: *Pneumocystis* pneumonia; PDN: prednisone; PE: plasma exchange; PRES: posterior reversible encephalopathy syndrome; PSL: prednisolone; RTX: rituximab; SAE: severe adverse event; s.c. subcutaneous; sJIA: systemic juvenile idiopathic arthritis; SLE: systemic lupus erythematosus; TAC: tacrolimus; TCZ: tocilizumab; TMA: thrombotic microangiopathy; TNF: tumor necrosis factor; UCTD: undifferentiated connective tissue disease; VCR: vincristine; VP16: etoposide; VZV: *Varicella-zoster* virus
