# Supplementary material for: Current treatment in macrophage activation syndrome worldwide: a systematic literature review to inform the METAPHOR project
Source: Rheumatology (Oxford). 2024 Jul 26;64(1):32–44. doi: 10.1093/rheumatology/keae391 (PMC11701305; doi:10.1093/rheumatology/keae391)
Supplement: keae391_Supplementary_Data [file keae391_supplementary_data.zip › keae391_Supplementary_Data/Supplementary_all responders.docx]

| **SURNAME** | **NAME** | **INSTITUTION** | **TOWN** | **COUNTRY** |
| --- | --- | --- | --- | --- |
| **Aalto** | Kristiina | Children's Hospital, Helsinki University Hospital | Helsinki | Finland |
| **Abud Mendoza** | Carlos | Hospital Central “Dr Ignacio Morones Prieto” | San Luis Potosí | Mexico |
| **Abushhaiwia** | Awatif | Tripoli Children's Hospital | Tripoli | Libya |
| **Ailioaie** | Constantin | Children Emergencies Hospital | Iasi | Romania |
| **Akikusa** | Jonathan | Royal Children's Hospital | Melbourne | Australia |
| **Aksu** | Guzide | Ege University Faculty of Medicine Deparment of Pediatrics | Izmir | Turkey |
| **Aktay Ayaz** | Nuray | Istanbul University Faculty of Medicine | Istanbul | Turkey |
| **Al Jashmi** | Ruqaiya Nasser | Oman Medical Speciality Board | Muscat | Oman |
| **Al-Abrawi** | Safiya | Royal Hospital | Muscat | Oman |
| **Alessio** | Maria | Università di Napoli Federico II | Napoli | Italy |
| **Alexeeva** | Ekaterina | State Institution Scientific Center of Children Health of Rams Moscow | Moscow | Russian Federation |
| **Al-Mayouf** | Sulaiman M. | King Faisal Specialist Hospital and Research Center | Riyadh | Saudi Arabia |
| **AlMutairi** | Abdulaziz | King Saud Medical City | Riyadh | Saudi Arabia |
| **Alsuweiti** | Muatasem | King Hussein Medical Center | Amman | Jordan |
| **Amalia** | Rizqi | Cipto Mangunkusumo Hospital | Jakarta | Indonesia |
| **AMIN** | Iman | Tripoli Children's Hospital | Tripoli | Libya |
| **Anton** | Jordi | Hospital Sant Joan de Déu, University of Barcelona | Esplugues de Llobregat, Barcelona | Spain |
| **Armbrust** | Wineke | Beatrix Kinderkliniek, University Medical Center | Groningen | Netherlands |
| **Astigarraga** | Itziar | Hospital de Cruces | Bilbao | Spain |
| **Bakkaloglu** | Sevcan | Gazi University Faculty of Medicine | Ankara | Turkey |
| **Bakry** | Reima | East Jeddah Hospital | Jeddah | Saudi Arabia |
| **Basaran** | Ozge | Hacettepe University Children's Hospital | Ankara | Turkey |
| **Basulto May** | Floricely | Instituto Mexicano Del Seguro Social | Coahuila | Mexico |
| **Bathia** | Jigna | Institute of Child Health | Kolkata | India |
| **Battagliotti** | Cristina | Hospital de Niños Dr Orlando Alassia | Santa Fe | Argentina |
| **Belot** | Alexandre | Hôpital Universitaire femme mère enfant | Bron (Lyon) | France |
| **Berg** | Stefan | The Queen Silvia Children's Hospital | Goteborg | Sweden |
| **Beutel** | Karin | Technische Universität München | München | Germany |
| **Bica** | Blanca | University Hospital Clementino Fraga Filho | Rio de Janeiro | Brazil |
| **Bolt** | Isabel | Berner Rheumazentrum | Bern | Switzerland |
| **Boyadzhiev** | Martin | Varna Medical University | Varna | Bulgaria |
| **Boyarchuk** | Oksana | Ternopil Regional Children's Hospital | Ternopil | Ukraine |
| **Boyko** | Yaryna | Western Ukrainian Specialized Children's Medical Centre | Lviv | Ukraine |
| **Bracaglia** | Claudia | Ospedale Pediatrico Bambino Gesù | Rome | Italy |
| **Breda** | Luciana | Ospedale Policlinico – Università degli studi di Chieti | Chieti | Italy |
| **Brochard** | Karine | Children's University Hospital | Toulouse | France |
| **Brogan** | Paul | Great Ormond St Hospital | London | United Kingdom |
| **Brunner** | Jurgen | Medical University Innsbruck | Innsbruck | Austria |
| **Bujan Rivas** | Segundo | Hospital Valle de Hebron | Barcelona | Spain |
| **Calin** | Aurelia | Medic Line Clinic | Bucarest | Romania |
| **Calzada** | Joan | Hospital Sant Joan de Déu, University of Barcelona | Esplugues de Llobregat, Barcelona | Spain |
| **Camacho Lovillo** | Marisol | University Hospital Virgen del Rocio | Seville | Spain |
| **Canna** | Scott | Children’s Hospital of Philadelphia | Philadelphia | United States |
| **Cannizzaro** | Elvira | Kinderspital Zürich | Zurich | Switzerland |
| **Caorsi** | Roberta | IRCCS Istituto Giannina Gaslini | Genova | Italy |
| **Carlomagno** | Raffaella | Centre Multisite Romand de Rhumatologie Pediatrique / Centre Hospitalier Universitaire Vaudois (CHUV) | Lausanne | Switzerland |
| **Cattalini** | Marco | Clinica Pediatrica dell'Università di Brescia, Spedali Civili | Brescia | Italy |
| **Celani** | Camilla | Ospedale Pediatrico Bambino Gesù | Rome | Italy |
| **Chan** | Kwai Yu Winnie | Queen Elizabeth Hospital | Hong Kong | Hong Kong |
| **Chandra** | Sharat | Cincinnati Children's Medical Center and University of Cincinnati | Cincinnati | United States |
| **Chandrakasan** | Shanmuganathan | Emory University School of Medicine | Atlanta, GA | United States |
| **Chang** | Chong U | Centro Hospitalar Conde de São Januário | Hong Kong | Hong Kong |
| **Chasnyk** | Vyacheslav | Saint-Petersburg State Pediatric Medical Academy | Saint-Petersburg | Russian Federation |
| **Chyzheuskaya** | Iryna | 4th city children's clinical hospital in Minsk | Minsk | Belarus |
| **Ciurtin** | Coziana | University College London | London | United Kingdom |
| **Clemente Garulo** | Daniel | University Hospital Niño Jesús | Madrid | Spain |
| **Cochino** | Alexis-Virgil | Institute for Mother and Child Care | Bucharest | Romania |
| **Consolaro** | Alessandro | IRCCS Istituto Giannina Gaslini | Genova | Italy |
| **Consolini** | Rita | Ospedale Santa Chiara, Università di Pisa | Pisa | Italy |
| **Cron** | Randy | University of Alabama at Birmingham | Birmingham, AL | United States |
| **da Silva** | Carlos Henrique M. | Hospital de Clinicas de Faculdades de Medicina da Universidade Federal de Uberlandia | Uberlandia | Brazil |
| **De Benedetti** | Fabrizio | Ospedale Pediatrico Bambino Gesù | Rome | Italy |
| **De Cunto** | Carmen | Buenos Aires Italy Hospital | Buenos Aires | Argentina |
| **De Matteis** | Arianna | Bambino Gesù Children’s Hospital | Rome | Italy |
| **De Somer** | Lien | University Hospital Gasthuisberg | Leuven | Belgium |
| **Dedeoglu** | Fatma | Children's Hospital Medical Center | Boston, MA | United States |
| **Deepakbabu** | Chellapandian | Johns Hopkins Hospital | Baltimora | United States |
| **Del Giudice** | Emanuela | Santa Maria Goretti Hospital, Polo Pontino, Sapienza University of Rome | Latina | Italy |
| **Diaz Maldonado** | Adriana Soraya | Consultorio privado - Bogota | Bogotà | Colombia |
| **Dolezalova** | Pavla | Charles University in Prague and General University Hospital | Praha | Czech Republic |
| **Dressler** | Frank | Hannover Medical School | Hannover | Germany |
| **Dzhus** | Marta | National Medical University Bohomlets, Alexander Clinical Hospital | Kiev | Ukraine |
| **El Miedany** | Yasser | Ain Shams University | Cairo | Egypt |
| **El-Ghoneimy** | Dalia | El-Demardash Children's Hospital | Cairo | Egypt |
| **Emminger** | Wolfgang | University Children's Hospital | Vienna | Austria |
| **Enciso** | Sandra | Hospital de la Beneficiencia española | Mexico City | Mexico |
| **Estmann** | Anne | Odense University Hospital | Odense | Denmark |
| **Etayari** | Hala | Tripoli Children's Hospital | Tripoli | Libya |
| **Fair** | Danielle | Medical College of Wisconsin | Milwaukee | United States |
| **Fasshauer** | Maria | Hospital St. Georg gGmbH Leipzig, affiliated to the University of Leipzig | Leipzig | Germany |
| **Fassi** | Daniel | Rigshospitalet | Copenhagen | Denmark |
| **Faugier** | Enrique | Hospital Infantil de México Federico Gómez | Mexico City | Mexico |
| **Federici** | Silvia | Ospedale Pediatrico Bambino Gesù | Rome | Italy |
| **Feldman** | Brian | University of Toronto, The Hospital for Sick Children | Toronto, ON | Canada |
| **Filocamo** | Giovanni | Fondazione IRCCS Ca' Granda-Ospedale Maggiore Policlinico | Milan | Italy |
| **Flato** | Berit | Oslo University Hospital and University of Oslo | Oslo | Norway |
| **Fluchel** | Mark | Seattle Children's Hospital | Seattle | United States |
| **Foell** | Dirk | University Hospital Muenster | Muenster | Germany |
| **Fotis** | Lampros | University General Hospital Attikon | Athens | Greece |
| **Frkovic** | Marijan | University Hospital Centre Zagreb, University School of Medicine | Zagreb | Croatia |
| **Fuhlbrigge** | Robert | Children's Hospital Colorado/ University of Colorado School of Medicine | Aurora | United States |
| **Gaggiano** | Carla | Policlinico le Scotte, Universita' di Siena | Siena | Italy |
| **Gagro** | Alenka | Children's Hospital Zagreb | Zagreb | Croatia |
| **Gallizzi** | Romina | Azienda Ospedaliera Universitaria Renato Dulbecco di Catanzaro | Catanzaro | Italy |
| **Garcia Cunha** | Ana Luiza | Children's hospital João Paulo II | Belo Horizonte | Brazil |
| **Garcia-Rodriguez** | Fernando | Hospital Universitario "Dr. Jose E. González", UANL | Monterrey | Mexico |
| **García-Rodríguez** | Fernando | Hospital San José; TecSalud | Monterrey, Nuevo Léon | Mexico |
| **Gattorno** | Marco | IRCCS Istituto Giannina Gaslini | Genova | Italy |
| **Girschick** | Hermann | Perinatal Centre of the Vivantes Klinikum | Berlin | Germany |
| **Glerup** | Mia | Aarhus University Hospital | Aarhus | Denmark |
| **Grebenkina** | Lyudmila | Togliatti City Clinical Hospital №5 | Togliatti | Russian Federation |
| **Guha** | Suparna | Vivekananda Institute of Medical Sciences | KOLKATA | India |
| **Gutierrez Suarez** | Raul | Hospital del niño. Instituto Materno Infantil del Estado de México (IMIEM) | Toluca | Mexico |
| **Guzman Ramirez** | Jaime | British Columbia Children's Hospital | Vancouver, BC | Canada |
| **Hadef** | Djohra | University hospital center of Batna | Batna | Algeria |
| **Hamad Saied** | Mohamad | Carmel Medical Centre | Haifa | Israel |
| **Hashad** | Soad | Tripoli Children's Hospital | Tripoli | Libya |
| **Hashkes** | Philip (Pinchas) | Shaare Zedek Medical Center | Jerusalem | Israel |
| **Hasle** | Henrik | Aarhus Universitetshospital | Aarhus | Denmark |
| **Hays** | Julia Allyson | Children’s Mercy Kansas City | Kansas City | United States |
| **Held** | Martina | University Hospital Centre Zagreb, University School of Medicine | Zagreb | Croatia |
| **Henter** | Jan-Inge | Karolinska University Hospital | Stockholm | Sweden |
| **Herrera** | Cristina N | Hospital de Niños Roberto Gilbert Elizalde | Guayaquil | Ecuador |
| **Heshin-Bekenstein** | Merav | Dana Children's Hospital Tel Aviv Medical Center | Tel Aviv | Israel |
| **Ho** | Assunta Chi Hang | Prince of Wales Hospital, Chinese University of Hong Kong | Hong Kong | Hong Kong |
| **Horne** | Anna Carin | Karolinska University Hospital | Stockholm | Sweden |
| **Horneff** | Gerd | Asklepios Children’s Hospital Sankt Augustin | Sankt Augustin | Germany |
| **Huang** | Jing-Long | Chang-Gung Memorial Hospital | Taoyuan | Taiwan, Province of China |
| **Hufnagel** | Markus | University Medical Center Freiburg | Freiburg | Germany |
| **Ilisson** | Jaanika | Tartu University Hospital, Children' s Clinic | Tartu | Estonia |
| **Islam** | Mohammad Imnul | Bangabandhu Sheikh Mujib Medical University (BSMMU) | Dhaka | Bangladesh |
| **Ivanova** | Viktoriia | Regional children hospital | Kirovograd | Ukraine |
| **Izawa** | Kazushi | Kyoto University Hospital | Kyoto | Japan |
| **Janda** | Ales | University Medical Center Ulm | Ulm | Germany |
| **Jelusic** | Marija | University Hospital Centre Zagreb, University School of Medicine | Zagreb | Croatia |
| **Jeong** | Daechul | Catholic University Of Korea | Seoul | Korea, Republic Of |
| **Jesudas** | Rohith | St Jude Children's Research Hospital | Memphis | United States |
| **Jindal** | Ankur | Postgraduate Institute of Medical Education and Research | Chandigarh | India |
| **Jiron mendiola** | Karla Vanessa | Hospital Infantil De Nicaragua | Managua | Nicaragua |
| **K. Oliveira** | Sheila | Universidade Federal do Rio de Janeiro | Rio de Janeiro | Brazil |
| **Kahn** | Robin | Lund University | Lund | Sweden |
| **Kaposzta** | Rita | University of Debrecen | Debrecen | Hungary |
| **Kasapcopur** | Ozgur | Istanbul University, Cerrahpasa Medical Faculty | Istanbul | Turkey |
| **Katsicas** | Maria Martha | Hospital de Pediatria Juan P. Garrahan | Buenos Aires | Argentina |
| **Kavirayani** | Akhila | Oxford University Hospitals NHS Foundation Trust | Oxford | United Kingdom |
| **Keenan** | Camille | Seattle Children's Hospital | Seattle | United States |
| **Khaosut** | Parichat | King Chulalongkorn Memorial Hospital | Bangkok | Thailand |
| **Khawaja** | Khulood | Sheikh Shakhbout Medical City | Abu Dhabi | United Arab Emirates |
| **Khedr** | Waleed Ahmed Salaheldeen Hassan | Benha Universty hospital - Benha children hospital | Benha | Egypt |
| **Khubchandani** | Raju | SRCC Childrens Hospital | Mumbai | India |
| **Kobusinska** | Katarzyna | Wojewódzki Szpital Dziecięcy im. J. Brudzińskiego | Bydgoszcz | Poland |
| **Koker** | Oya | Sisli Hamidiye Etfal Research and Training Hospital | Istanbul | Turkey |
| **Koné-Paut** | Isabelle | Le Kremlin-Bicêtre University Hospital, Paris-Sud University - CEREMAI | Le Kremlin Bicêtre (Paris) | France |
| **Kostik** | Mikhail | Saint-Petersburg State Pediatric Medical Academy | Saint-Petersburg | Russian Federation |
| **Kuemmerle-Deschner** | Jasmin | University Children’s Hospital Tuebingen | Tuebingen | Germany |
| **Kumar** | Ashish | Cincinnati Children's Medical Center and University of Cincinnati | Cincinnati | United States |
| **La Rosée** | Paul | Schwarzwald-Baar-Klinikum | Villingen-Schwenningen | Germany |
| **Ladino Ramirez** | Mabel Aurora | Hospital San Juan de Dios | Santiago | Chile |
| **Lazar** | Calin | Children Emergencies Hospital | Cluj-Napoca | Romania |
| **Lee** | Chongwei | Tianjin Children's Hospital | Tianjin | China |
| **Lehmann** | Hartwig | University Hospital Giessen | Giessen | Germany |
| **Lehmberg** | Kai | University Hospital Hamburg-Eppendorf | Hamburg | Germany |
| **Li** | Caifeng | Beijing Children's Hospital, Capital | Beijing | China |
| **Li** | Xiaoqing | Xi'an Children's Hospital | Xi'an,Shaanxi province | China |
| **Licciardi** | Francesco | Paediatrics, University of Torino | Torino | Italy |
| **Lima** | Joana | University Hospital Oporto | Oporto | Portugal |
| **Lorenz** | Zoref | Cincinnati Children's Hospital Medical Center | Cincinnati, OH | United States |
| **Lotfy** | Hala | Cairo University Paediatric Hospital | Cairo | Egypt |
| **Lovell** | Daniel J. | Cincinnati Children's Hospital Medical Center | Cincinnati, OH | United States |
| **Lu** | Meiping | Children's hospital of Zhejiang University School of Medicine | Hangzhou | China |
| **Lukjanoviča** | Kristīne | University Children Hospital | Riga | Latvia |
| **Maggio** | Maria Cristina | Children Hospital "G. Di Cristina" | Palermo | Italy |
| **Magni-Manzoni** | Silvia | Ospedale Pediatrico Bambino Gesù | Rome | Italy |
| **Maher** | Sheren esam maher | Minia University Hospital | Minia | Egypt |
| **Majeed** | Mahmoud | King Fahad Armed Forces Hospital | Jeddah | Saudi Arabia |
| **Maritsi** | Despoina | Athens Medical School, University of Athens | Athens | Greece |
| **Marsh** | Rebecca | Cincinnati Children's Medical Center and University of Cincinnati | Cincinnati | United States |
| **Martini** | Giorgia | Presidio Ospedaliero Universitario "Santa Maria della Misericordia" | Udine | Italy |
| **Masmas** | Tania Nicole | Rigshospitalet | Copenhagen | Denmark |
| **Mastrolia** | Maria Vincenza | Azienda Ospedaliero Universitaria Meyer | Firenze | Italy |
| **Mauro** | Angela | Fatebenefratelli Hospital | Milano | Italy |
| **Mehregan** | Fatemeh Feresteh | Loghman hakim Hospital | Teheran | Iran, Islamic Republic of |
| **Mejbri** | Manel | Lausanne University Hospital (CHUV) | Lausanne | Switzerland |
| **Melki** | Isabelle | Université Paris-Cité, IMAGINE Institute, Necker Children’s Hospital | Paris | France |
| **Miettunen** | Paivi | Alberta Children's Hospital | Calgary | Canada |
| **Miniaci** | Angela | Azienda Ospedaliero-Universitaria S.Orsola-Malpighi | Bologna | Italy |
| **Minoia** | Francesca | Fondazione IRCCS Ca' Granda-Ospedale Maggiore Policlinico | Milan | Italy |
| **Mizuta** | Mao | Tokyo Medical and Dental University (TMDU) | Tokyo | Japan |
| **Mondal** | Rakesh | Medical College Kolkata, Kolkata | Kolkata | India |
| **Montin** | Davide | Paediatrics, University of Torino | Torino | Italy |
| **Morel Ayala** | Zoilo | Centro Materno Infantil. Hospital De Clinicas. Universidad Nacional De Asuncion. | San Lorenzo | Paraguay |
| **Mosquera Angarita** | Juan Manuel | Hospital Sant Joan de Déu, University of Barcelona | Esplugues de Llobregat, Barcelona | Spain |
| **Mukusheva** | Zaure | Corporate Fund “University Medical Center” National Research Center for Maternal and Child Health | Astana | Kazakhstan |
| **Myrup** | Charlotte | Rigshospitalet | Copenhagen | Denmark |
| **Nakhutsrishvili** | Eka | Tbilisi State Medical University Pediatric Clinic | Tbilisi | Georgia |
| **Naqvi** | Ahmed | The Hospital for Sick Children | Toronto | Canada |
| **Narazaki** | Hidehiko | Nippon Medical School Hospital | Tokyo | Japan |
| **Nascimento** | Joao | Hospital Pediatrico Coimbra | Braga | Portugal |
| **Navarrete** | Carmen | Roberto del Rio Children`s Hospital | Santiago | Chile |
| **Nordal** | Ellen Berit | University Hospital of Northern Norway | Tromso | Norway |
| **Ogbu** | Ekemini A | Cincinnati Children's Medical Center and University of Cincinnati | Cincinnati | United States |
| **Ogunjimi** | Benson | Queen Paola Hospital ZNA | Antwerp | Belgium |
| **Okong'o** | Lawrence Owino | Kenyatta National Hospital | Nairobi | Kenya |
| **Oliveira-Ramos** | Filipa | Hospital Santa Maria | Lisbon | Portugal |
| **Omenetti** | Alessia | Ospedale Pediatrico G. Salesi di Ancona | Ancona | Italy |
| **Opoka-Winiarska** | Violetta | Medical University of Lublin | Lublin | Poland |
| **Orlando** | Francesca | Universita' di Napoli Federico II | Napoli | Italy |
| **Ozdemir Cicek** | Sumeyra | Erciyes University | Kayseri/Melikgazi | Turkey |
| **Ozen** | Seza | Hacettepe University Children's Hospital | Ankara | Turkey |
| **Pain** | Clare | Alder Hey Children's NHS Foundation Trust | Liverpool | United Kingdom |
| **Pal** | Priyankar | Institute of Child Health | Kolkata | India |
| **Palmou Fontana** | Natalia | Hospital De Valdecilla | Santander | Spain |
| **Papadopoulou** | Charalampia | Great Ormond St Hospital | London | United Kingdom |
| **Pardeo** | Manuela | Ospedale Pediatrico Bambino Gesù | Rome | Italy |
| **Petrovic** | Gordana | Mother and Child Health Institute | Belgrade | Serbia |
| **Picarelli** | Mercedes | Pontificia Universidade do Rio Grande do Sul | Porto Alegre | Brazil |
| **Pilania** | Rakesh Kumar | Postgraduate Institute of Medical Education and Research | Chandigarh | India |
| **Pilkington** | Clarissa | Great Ormond St Hospital | London | United Kingdom |
| **Pinedo** | Maria del Carmen | Hospital de Cruces | Bilbao Vizcaya | Spain |
| **Pratsidou-Gertsi** | Polyxeni | Hippokration General Hospital, Thessaloniki University School of Medicine | Thessaloniki | Greece |
| **Pruunsild** | Chris | Tartu University Hospital, Children' s Clinic | Tartu | Estonia |
| **Quarmyne** | Maa-Ohui | Phoenix Children's Hospital | Phoenix | United States |
| **Quartier** | Pierre | Université Paris-Cité, IMAGINE Institute, Necker Children’s Hospital | Paris | France |
| **Raees Karami** | Seyed Reza | Imam Khomeini Hospital, Vali-e-Asr | Teheran | Iran, Islamic Republic of |
| **Ramme** | Kim | Akademiska sjukhuset | Akademiska, Uppsala | Sweden |
| **Ravelli** | Angelo | IRCCS Istituto Giannina Gaslini | Genova | Italy |
| **Razanamahery** | Jerome | CHU Dijon Bourgogne | Dijon | France |
| **Rebane** | Katariina | Children's Hospital, Helsinki University Hospital | Helsinki | Finland |
| **Remesal** | Agustin | University Hospital La Paz | Madrid | Spain |
| **Retornaz** | Karine | CHU Nord | Marseille | France |
| **Rigante** | Donato | Cattolica Sacro Cuore University | Roma | Italy |
| **Rocco** | Joseph | National Institute of Allergy and Infectious Disease (NIH) | Bethesda | United States |
| **Rodrigues Fonseca** | Adriana | Universidade Federal do Rio de Janeiro | Rio de Janeiro | Brazil |
| **Rodriguez Lozano** | Ana Luisa | Instituto Nacional de Pediatria | Mexico City | Mexico |
| **Rodriguez-Aguayo** | Sandra | Hospital Infantil de México Federico Gomez | Mexico City | Mexico |
| **Rojas** | Jorge | Hospital Dr. Exequiel Gonzalez Cortes | Santiago | Chile |
| **Rossano** | Martina | Fondazione IRCCS Ca' Granda-Ospedale Maggiore Policlinico | Milan | Italy |
| **Ryhanen** | Samppa | Helsinki University Hospital | Helsinki | Norway |
| **Saad Magalhaes** | Claudia | Hospital das Clinicas - Botucatu Medicine University, UNESP | Botucatu | Brazil |
| **Sadeghi** | Payman | Bahrami Children's Hospital | Tehran | Iran, Islamic Republic of |
| **Sag** | Erdal | Hacettepe University Children's Hospital | Ankara | Turkey |
| **Saldana Davila** | Blachy Javier | Children's National Hospital | Washington | United States |
| **Salehzadeh** | Farhad | Bouali children's Hospital | Ardabil | Iran, Islamic Republic of |
| **Sánchez-Manubens** | Judith | Hospital de Sabadell | Barcelona | Spain |
| **Sawhney** | Sujata | Sir Ganga Ram Hospital Marg | New Delhi | India |
| **Schulert** | Grant | Cincinnati Children's Hospital Medical Center | Cincinnati, OH | United States |
| **Schvartz** | Adrien | Le Kremlin-Bicêtre University Hospital, Paris-Sud University - CEREMAI | Le Kremlin Bicêtre (Paris) | France |
| **Selcuk** | Yuksel | Pamukkale University School of Medicine | Denizli | Turkey |
| **Selmanovic Mulaosmanovic** | Velma | Children's Hospital University Clinical Center Sarajevo | Sarajevo | Bosnia and Herzegovina |
| **Sen** | Ethan | Bristol Royal Hospital for Children Royal National Hospital for Rheumatic Diseases | Bristol | United Kingdom |
| **Sener** | Seher | Hacettepe University Children's Hospital | Ankara | Turkey |
| **Shanbhag Mohite** | Rachna | Aster CMI Hospital | Bangalore | India |
| **Sharma** | Avinash | Dr Rajendra Prasad Government Medical College, Tanda | Kangra | India |
| **Shehata** | Rawia Salama | Maternity children hospital | Jeddah | Saudi Arabia |
| **Shimizu** | Masaki | Tokyo Medical and Dental University (TMDU) | Tokyo | Japan |
| **Simonini** | Gabriele | Azienda Ospedaliero Universitaria Meyer | Firenze | Italy |
| **Singh** | Surjit | Postgraduate Institute of Medical Education and Research | Chandigarh | India |
| **Smerla** | Roubini | Paidon Hospital | Athens | Greece |
| **Šnipaitienė** | Aušra | Hospital of Lithuanian University of Health Sciences Kauno Klinikos | Kaunas | Lithuania |
| **Sobh** | Ali | Mansoura University Children's Hospital | Mansura | Egypt |
| **Sozeri** | Betul | Health Sciences University, Umraniye Education and Research Hospital | Istanbul | Turkey |
| **Sparchez** | Mihaela | Children Emergencies Hospital | Cluj-Napoca | Romania |
| **Sršen** | Saša | Universitiy hospital centre Split | Split | Croatia |
| **Stanevicha** | Valda | University Children Hospital | Riga | Latvia |
| **Swart** | Joost | Wilhelmina Kinderziekenhuis | Utrecht | Netherlands |
| **Sztajnbok** | Flavio | University Hospital Pedro Ernesto | Rio de Janeiro | Brazil |
| **Tangcheewinsirikul** | Sirikarn | Faculty of Medicine Vajira Hospital, Navamindradhiraj University | Bangkok | Thailand |
| **Temelkova** | Katya | University Children Hospital | Sofia | Bulgaria |
| **Tenbrock** | Klaus | RWTH Aachen University Hospital | Aachen | Germany |
| **Toplak** | Natasa | University Children's Hospital, University Medical Centre Ljubljana | Ljubljana | Slovenia |
| **Torno** | Lilibeth | Children Hospital of Orange County | Orange (CA) | United States |
| **Trevisan** | Matteo | IRCCS Ospedale Pediatrico Bambino Gesù | Roma | Italy |
| **Tsinti** | Maria | University of Athens Medical School, Children Hospital Aghia Sophia | Athens | Greece |
| **Tsitsami** | Elena | University of Athens Medical School, Children Hospital Aghia Sophia | Athens | Greece |
| **Twilt** | Marinka | Alberta Children's Hospital | Calgary | Canada |
| **Van den Berg** | J.Merlijn | Emma Children Hospital, Academic Medical Center (AMC) | Amsterdam | Netherlands |
| **van Laar** | Jan A.M. | Erasmus MC | Rotterdam | Netherlands |
| **Vargas Rincon** | Camilo Andres | Saludcoop Cali | Cali | Colombia |
| **Varnier** | Giulia Camilla | Royal Manchester Children's Hospital | Manchester | United Kingdom |
| **Vastert** | Sebastiaan | Wilhelmina Kinderziekenhuis | Utrecht | Netherlands |
| **Vega Cornejo** | Gabriel | Hospital México Americano | Guadalajara | Mexico |
| **Verdoni** | Lucio | ASST Papa Giovanni XXIII | Bergamo | Italy |
| **Viola** | Diego Oscar | Instituto CAICI | Rosario | Argentina |
| **Vojinovic** | Jelena | Clinical Center, Faculty of Medicine, University of Nis | Nis | Serbia |
| **Vyzhga** | Yulia | National Pirogov Memorial Medical University | Vinnytsia | Ukraine |
| **Wahlin** | Bjorn | Karolinska Institutet | Stockholm | Sweden |
| **Wakiguchi** | Hiroyuki | Yamaguchi University Graduate School of Medicine | Ube | Japan |
| **Weiser** | Peter | AWIMED S.R.O. | Staré Mesto | Slovakia |
| **Wiesik-Szewczyk** | Ewa | Central Clinical Hospital of the Ministry of National Defense, Military Institute of Medicine | Warsaw | Poland |
| **Yamazaki** | Kazuko | St. Marianna University School of Medicine | Kawasaki | Japan |
| **Yasumura** | Junko | JR Hiroshima Hospital | Hiroshima | Japan |
| **Yin** | Wei | Wuhan Children’s hospital, Tongji Medical college, Huazhong University of Science & Technology | Wuhan | China |
| **Zeng** | Huasong | Guangzhou Women and Children’s Medical Center | Guangzhou | China |
| **Zhang** | Wei | Chengdu Women’s and Children’s Central Hospital | Chengdu | China |
| **Ziaee** | Vahid | Children's Hospital, Medical Center | Teheran | Iran, Islamic Republic of |
| **Ziv** | Amit | Meir Medical Centre | Kfar Saba | Israel |
| **Zuber** | Zbigniew | St Louis Children's Hospital | Cracow | Poland |
